# Immune Intervention in Sepsis

**DOI:** 10.3389/fphar.2021.718089

**Published:** 2021-07-14

**Authors:** Jian Chen, Haiming Wei

**Affiliations:** ^1^ Department of Intensive Care Medicine, The First Affiliated Hospital of, USTC, Division of Life Science and Medicine, University of Science and Technology of China, Hefei, China; ^2^ Department of Geriatrics, The First Affiliated Hospital of, USTC, Division of Life Science and Medicine, University of Science and Technology of China, Hefei, China; ^3^ Institute of Immunology, University of Science and Technology of China, Hefei, China

**Keywords:** sepsis, inflammatory, cytokine, immunosuppression, immune intervention

## Abstract

Sepsis is a host immune disorder induced by infection. It can lead to multiple organ dysfunction syndrome (MODS), which has high morbidity and mortality. There has been great progress in the clinical diagnosis and treatment of sepsis, such as improvements in pathogen detection technology, innovations regarding anti-infection drugs, and the development of organ function support. Abnormal immune responses triggered by pathogens, ranging from excessive inflammation to immunosuppression, are recognized to be an important cause of the high mortality rate. However, no drugs have been approved specifically for treating sepsis. Here, we review the recent research progress on immune responses in sepsis to provide a theoretical basis for the treatment of sepsis. Constructing and optimizing a dynamic immune system treatment regimen based on anti-infection treatment, fluid replacement, organ function support, and timely use of immunomodulatory interventions may improve the prognosis of sepsis patients.

## Introduction

Sepsis is a life-threatening syndrome caused by an abnormal infection-induced immune response. It is frequently seen in cases of severe infection, trauma, burns, shock, and major surgery. Sepsis can further develop into multiple organ dysfunction syndrome (MODS), which is the primary cause of death among acute and critically ill patients (Rhee et al., 2017). In recent years, the incidence rate of sepsis has increased globally, severely threatening human health and posing a tremendous burden on the economy and society (Adhikari et al., 2010; Fleischmann et al., 2016; Xie et al., 2020). Nowadays, it is thought that sepsis is mainly induced by immune dysfunction. To be specific, it develops from an initial excessive inflammatory response specific to pathogenic factors (such as infection or trauma) into immune paralysis or immunosuppression. In the excessive inflammatory response stage, the innate immune response, which should play a defensive role, causes cell and tissue injury, or even MODS (van der et al., 2017; Muszynski der et al., 2018).

**FIGURE 1:**
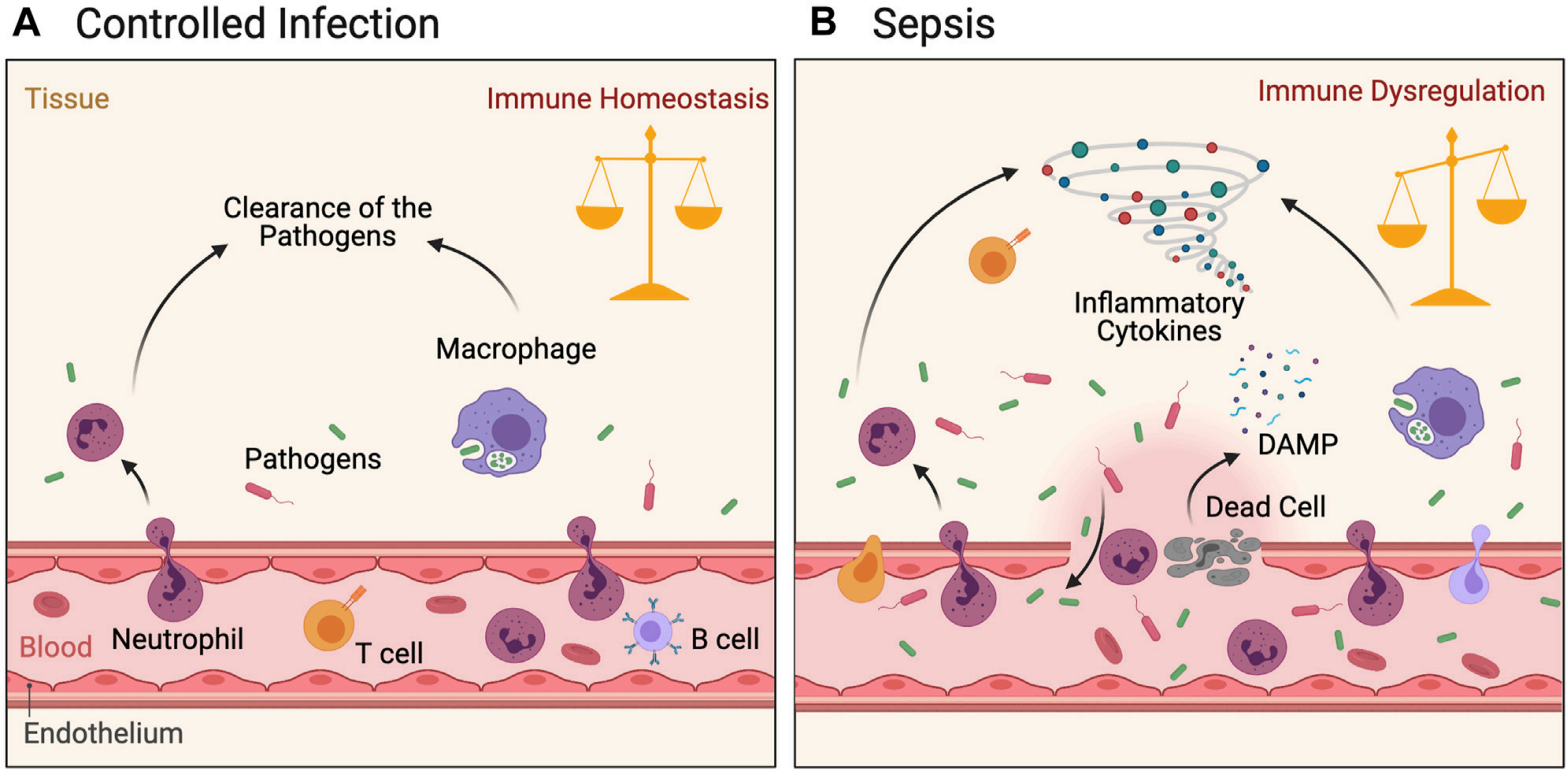
Immune response following pathogen infection. Figure **(A)**: Pathogen invasion induces local inflammatory response in tissues. The activated innate immune and adaptive immune cells migrate locally to tissues, inhibit microbe duplication and systematic dissemination, and finally clear the pathogen. Inflammation is controlled and immune balance of the body is achieved. **(B)**: Heavy load infection accompanied by local damage activated innate and adaptive immune cells are in a hyperinflammatory state under the dual effects of pathogens and DAMPs. The cytokine storm generated by these cells inhibits the pathogens to a certain extent, but also leads to further tissue damage. Microbes migrate to the whole body through damaged blood vessels, causing a strong inflammatory response and thus leading to systemic immune dysregulation and injury.

The understanding of sepsis has gone through three stages. In the first stage, the concept of systemic inflammatory response syndrome (SIRS) was proposed (Bone et al., 1992), based on the hypothesis that sepsis is an infection-induced systemic inflammatory response. However, there was debate concerning this concept, with a focus on the specificity of the diagnosis. The low specificity of the SIRS diagnostic criteria made the patient population that fulfilled the diagnostic criteria for sepsis extremely complicated, which caused great difficulties in clinical research. In the second stage, to improve the diagnostic specificity, it was proposed that the diagnostic criteria for sepsis should not just be restricted to an inflammatory response. Instead, more attention should be paid to hemodynamics, tissue perfusion, and organ functional status. However, the new diagnostic criteria were not extensively applied in clinical practice due to their complexity (Levy et al., 2003). In the third and current stage, The Third International Consensus Definition for Sepsis and Septic Shock (Sepsis-3) were proposed (Singer et al., 2016). From the perspective of immunology, the essence of sepsis is MODS induced by a disordered immune response to severe infection, and the key factors include not only a systemic inflammatory response, but also disordered immune regulation (Figure 1B). In other words, sepsis develops from excessive immune activation into extensive immunosuppression (van der et al., 2017). This review explores the latest progress regarding the understanding of the pathogenesis of infectious sepsis-related immune dysfunction, so as to provide more theoretical evidence for developing new sepsis treatments. Immunity dysfunction caused by other causes such as trauma, major surgery or shock will be explored in the future.

## Pathogens and Immunity

### Bacteria

The host’s innate and adaptive immune responses are activated during bacterial infection, which is an important mechanism for resisting pathogen infection. In the early stage of a bacterial infection, the innate immune system is rapidly activated to locally restrict the infection *via* inflammatory responses to prevent further progression into systemic infection (Figure 1A). The innate immune system resists the invading pathogenic bacteria using immunologically active substances such as lysozyme and antibacterial peptides. Furthermore, during infection, the family of complement molecules in the blood can infiltrate the infected tissues and exert antibacterial effects *via* three complement activation pathways. If the infection persists, the infected cells can recruit innate immune cells such as neutrophils and monocytes in the blood by releasing chemokines such as C-X-C motif chemokine ligand 1 (CXCL1) and C-C motif ligand 8 (CCL8). These cells migrate from the blood vessels to the local tissue to exert an inflammatory effect. Toll-like receptors (TLRs) are important receptors associated with innate immunity. They specifically recognize and bind to pathogen-associated molecular patterns (PAMPs) (Pradeu and Cooper, 2012; Netea et al., 2017), triggering a series of signaling pathways that leads to inflammatory factor release and ultimately activates the adaptive immune system (Fitzgerald et al., 2003; Kawai and Akria, 2010).

**FIGURE 2:**
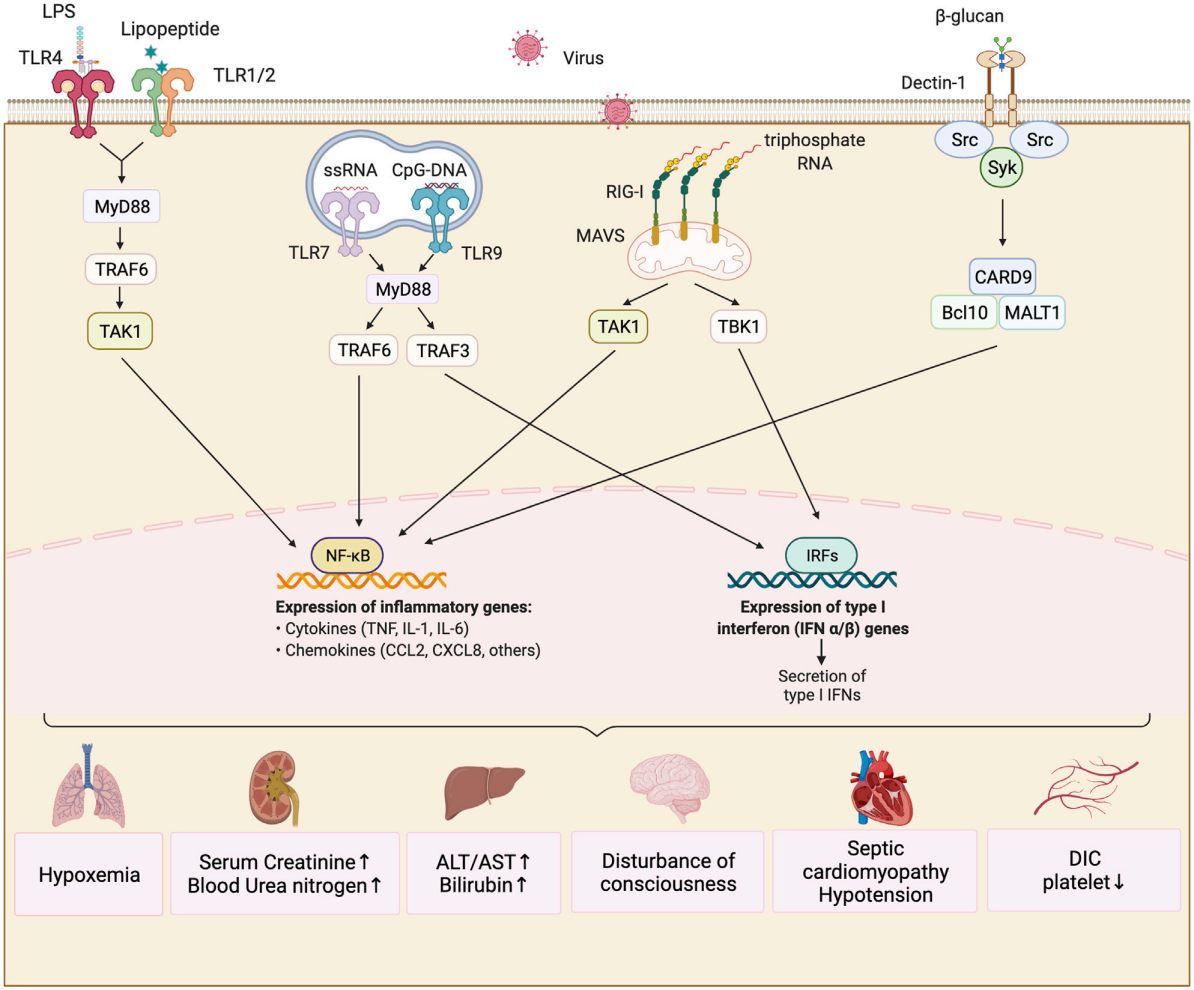
Immunity mechanism of sepsis induced by different pathogens. The predominant pathogens that cause sepsis are bacteria, fungi and viruses. The lipopolysaccharide (LPS) in the outer membrane of Gram-negative bacteria can be recognized by TLR4, while the lipoprotein in the cell wall of Gram-positive bacteria can be bound to TLR2/TLR1. They activate the downstream signaling molecule of MyD88 and ubiquitinate TRAF6. Ubiquitinized TRAF6K recruits the TAK1-TAB1/2 protein complex, while TAK1 kinase activates the transcription factor NF-kB and facilitates the production of key pro-inflammatory cytokines such as IL-1, IL-6, and TNF. Fungus β-glucan and mannose are commonly recognized by CLRs which mobilizes Syk protein kinases to coordinate the innate immune response, and eventually activate NFkB to produce pro-inflammatory factors through the CARD9/BCL10/MALT1 complex. iIfluenza viruse causing common viral infection in sepsis can be recognized by RIG-I in the cytoplasm and signal to TAK1 and TBK1 through oligomerization of MAVs molecules on mitochondria, which ultimately activates NF-kB and IRF family transcription factors and promotes the production of inflammatory factors and type 1 interferon. These changes cause damage to important viscera of airframe.

Regarding Gram-negative bacteria (Raetz and Whitfielg, 2002), lipopolysaccharide (LPS) in their outer membrane can be recognized by TLR4 (Figure 2). After recognition of LPS, TLR4 undergoes dimerization and further activates downstream signals, including myeloid differentiation factor 88 (MYD88) and MYD88 adaptor-like (MAL). MYD88 can recruit the downstream kinases interleukin-1 receptor-associated kinase 1(IRAK1), IRAK4, and tumor necrosis factor receptor-associated factor 6 (TRAF6), and ubiquitinate TRAF6. The ubiquitinated TRAF6 can recruit Transforming growth factor-β-activating kinase 1 (TAK1) TAK1 binding protein 1 and 2 (TAB1/2) complexes by acting as a scaffold molecule, and then TAK1 can activate IκB kinase (IKK) β of the IKK complex. The activated IKKβ phosphorylates the inhibitory molecule IkB of nuclear factor (NF)-κB in the cytoplasm, undergoes ubiquitination followed by degradation, and causes activated NF-κB to localize to the nucleus (Ghosh and Karin, 2002). This promotes the production of key pro-inflammatory cytokines such as interleukin (IL)-1β, IL-6, and tumor necrosis factor (TNF)-α (Vaure and Liu, 2014). These core inflammatory factors can act on vascular endothelial cells to increase blood vessel permeability to further promote immune cell migration to sites of inflammation. They can also inhibit bacterial growth by increasing the body temperature to achieve infection control. In addition, LPS can induce the transcription of type-1 interferon (*IFN*) (O’Neill et al., 2013) and IFN-related genes (Yamamoto et al., 2002) downstream of TLR4 *via* a MYD88-independent signaling pathway, thus promoting antiviral and antibacterial effects.

Similarly, the lipoprotein component of the cell wall of Gram-positive bacteria or *mycoplasma* can be recognized by TLR2/TLR1 or TLR2/TLR6, which activate the NF-κB transcription factor *via* the same signaling pathway to induce inflammatory responses (Figure 2). Other members of the TLR family, such as TLR3, TLR7, TLR8, and TLR9, can recognize the nucleic acid components of viruses and bacteria. The activation of these receptors induces the production of pro-inflammatory cytokines such as IL-1β, IL-6, TNF-α, and type I IFN. Take TLR3 as an example (Zhang et al., 2013). The TRIF protein downstream of TLR3 signaling can recruit the E3 ubiquitin ligase TRAF3 and further activate TANK binding kinase 1 (TBK1) (Takeuchi and Akira, 2010). The activated TBK1 can phosphorylate the IRF3 transcription factor, which promotes type I IFN production after localizing to the nucleus. The activation of NF-κB and IRF3 can lead to a strong early inflammatory response in patients with sepsis. If the infection cannot be eliminated completely, the continuous high-intensity inflammatory response damages cells such as vascular endothelial cells, resulting in abnormal blood coagulation and impaired organ function.

Infection can cause tissue damage and the release of damage-associated molecular patterns (DAMPs). These are similar to PAMPs, although DAMPs (Peiseler and Kubes, 2018) are mostly endogenous proteins or nucleic acid molecules, such as high mobility group box 1 (HMGB1) and mitochondrial DNA. They can be recognized by innate immune cells after entering the cytoplasm or extracellular space. Therefore, DAMP release is another mechanism by which pathogens can affect the host’s immune response (Gong et al., 2020). Once released into the extracellular space, DAMPs can be sensed by afferent nerves, followed by transmission of signals to the spleen and other organs *via* the vagus nerve, involving acetylcholine release. Acetylcholine can bind to cholinergic receptors on the surface of macrophages and inhibit the release of pro-inflammatory cytokines.

### Fungi

Many studies have confirmed that C-type lectin receptors (CLRs) play a major role in the identification of β-dextran and mannosan of pathogenic fungi. When dendritic cells (DCs) respond to *Candida albicans* infection, spleen tyrosine kinase (Syk) is recruited by CLRs to coordinate the innate immune response (rather than MYD88, which is the downstream linker molecule of TLRs) (Whitney et al., 2014). CLRs include Dectin-1, Dectin-2, Dectin-3, Mannose receptor, Mincle, and DC-specific intercellular adhesion molecule-3-grabbing non-integrin (DC-SING), which are mainly expressed in the bone marrow and epithelial cells (Figure 2). Dectin-1 and Dectin-2, as pattern recognition receptors (PRRs), can sense *Candida albicans* and mycelium infections (Bain et al., 2021). Upon ligand binding to these receptors, signaling pathways such as NF-κB, mitogen-activated protein kinase (MAPK), and Calcium/calmodulin-dependent protein kinase II (CaMKII) are activated *via* Syk, which plays an important role in balancing the immune response, inflammatory reactions, and fungal infection response (Gringhuis et al., 2011). Dectin-1 does not rely on Ca^2+^ to recognize fungal β-dextran, while Dectin-2 relies on Ca^2+^ to recognize fungal α-mannosan. Moreover, Dectin-1 can activate the NF-κB signaling pathway by activating all subunits of NF-κB, while Dectin-2 can activate this pathway only by activating the c-REL subunit of NF-κB. Dectin-3 can recognize α-mannosan on fungal cell walls, and it plays an important role in the immune response to *Candida albicans*, *Blastomyces dermatitidis*, and other pathogens (Lobato and Pascual, 2013). It plays a synergistic role with Dectin-2 in the identification of α-mannosan of pathogenic fungi (Zhu et al., 2013).

CLRs respond to infection in a process that involves complex synergy among the CLRs (Drummond and Lionakis, 2016). However, it should be noted that the synergistic mechanism among CLRs has not been thoroughly studied (Ostrop and Lang, 2017). Additionally, CLRs play synergistic roles with other receptors. For example, TLR2 and TLR4 can also recognize glucuronic acid and mannosan of *Cryptococcus* (Gibson and Johnston, 2015; Leopold et al., 2016). However, the synergistic mechanism of CLRs and TLRs remains unclear.

When an invasive fungal infection develops further, the body activates adaptive immunity to defend against the infection. Antigen-presenting cells (APCs) initiate the adaptive immune response by presenting antigens to T cells. Both CD4^+^ and CD8^+^ T cells are involved in the response to fungal infection (Leopold et al., 2016; Marcos et al., 2016). The role of CD4^+^ T cells is the most important out of the two. In response to antigen stimulation, CD4^+^ T cells are activated, proliferate, and differentiate into helper T cells (Th cells). Under the influence of cytokines in the local immune microenvironment, Th cells differentiate into Th1, Th2, Th17, or regulatory T cells (Treg cells).

Th1 cells directly or indirectly secrete γ-IFN and IL family members (IL-2, IL-6, and IL-12) to promote the activation and proliferation of macrophages, natural killer (NK) cells, and CD8^+^ T cells. The fungi are then killed, including by phagocytosis (Geginat et al., 2014; Murdock et al., 2014). Th2 and Th17 cells are necessary for the response to mucous membrane candidiasis (Conti and Gaffen, 2015; Fei et al., 2015). IL family members (such as IL-4 and IL-10) secreted by Th2 cells can promote the proliferation of B cells and the production of antibodies, which mediate the humoral immune response. Moreover, Th2 cells secrete IL-13 and transforming growth factor (TGF)-β, which regulate the immune balance. For example, IL-13 can inhibit macrophage activation by IFN-γ, and TGF-β can inhibit neutrophil activation and proliferation. Th17 cells also play an important role during fungal infections. However, the mechanism of action still needs to be studied in depth (Fei et al., 2015). Treg cells have important functions regarding immune tolerance and maintaining homeostasis. Furthermore, they play a role in resisting microbial infections, but the mechanisms remain unclear (Shafiani et al., 2010; Leopold et al., 2016). When stimulated by antigens, B cells are activated, proliferate, and differentiate into B2 cells with the help of Treg cells. They then form a germinal center and produce specific antibodies to mediate the humoral immune response.

### Viruses

During viral sepsis, PRRs recognize specific endogenous or exogenous ligands (Takeuchi and Akira, 2010) and then trigger non-specific innate immunity, which involves producing pro-inflammatory cytokines and chemotactic factors (such as TNF-α, IL-1β, IL-12, and IL-18) and recruiting phagocytes. Additionally, PRRs can trigger adaptive immunity and locally activate the complement and coagulation systems (Takeuchi and Akira, 2010; van et al., 2017; Iwasaki and Medzhitov, 2004).

Influenza viruses infecting alveolar cells, macrophage, and DCs can be recognized by TLRs (TLR 3, TLR4, and TLR7). The resultant NF-κB signaling upregulates pro-inflammatory cytokines (such as TNF-α, IL-1β, IL-6, and IL-8) that damage the epithelial–endothelial barrier (Short et al., 2014) (Figure 2). Herpes simplex virus produces a strong response through the TLR2 and TLR9 signaling pathways. The IL-6 level influences the survival rate associated with herpes simplex encephalitis (Bociąga Jasik, et al., 2011). In addition, herpes simplex virus can increase the ratio of IL-8 to TNF-α in newborns . Human enterovirus infection is characterized by a type I IFN response, induced by PRRs that respond to RNA viruses. The characteristic enterovirus infection response, which is a type I IFN response, mainly involves RIG-I-like receptor (RLR) signaling and TLR signaling [among the TLRs, TLR3, TLR7, TLR8, and TLR9 are related to nonspecific immune responses (Triantafilou et al., 2005; Richer et al., 2009; Coyne et al., 2011; Hsiao et al., 2014)]. Enterovirus infection not only causes pro-inflammatory cytokine release, but also induces cell death, thereby aggravating the inflammatory reaction. Dengue virus can also cause inflammatory reactions *via* the type I IFN response. In SARS-CoV infection, a high initial viral titer is related to extensive lung injury. Increases in monocyte, macrophage, and pulmonary neutrophil infiltration, serum pro-inflammatory cytokines (such as TNF-α, IL-6, IL-8, and IFN-γ), and chemotactic factors may be related to poor prognosis of Severe Acute Respiratory Syndrome Coronavirus (SARS-CoV) infection (Wong et al., 2004; Kong et al., 2009). Thus, sepsis related to SARS is associated with the direct cytopathic effect and cytokine storm caused by SARS-CoV. Middle East Respiratory Syndrome Coronavirus (MERS-CoV) can infect immunocytes such as DCs and macrophages and induce the production of pro-inflammatory cytokines and chemotactic factors, such as TNF-α, IL-6, CXCL-10, CCL-2, CCL-3, CCL-5, and IL-8 (Wong et al., 2004; Kong et al., 2009). Recent research has shown that the levels of pro-inflammatory cytokines and chemotactic factors in coronavirus disease 2019 (COVID-19) patients are significantly increased (Chu et al., 2014; Zhou et al., 2014; Huang et al., 2020; Liu et al., 2020).

### Immune Dysregulation During Sepsis

The pathogenesis of immune dysfunction in sepsis remains unclear. However, as early as in 1996, the American researcher Bone (1996) proposed that the development of sepsis is induced by an imbalance between the pro- and anti-inflammatory mechanisms in the body. Previous opinion maintained that the development of sepsis could be classified as an early systemic inflammatory response stage sequentially followed by a later compensatory anti-inflammatory response stage (Hotchkiss et al., 2013; Leentjens et al., 2013). However, since 2000, the hypothesis regarding SIRS sequentially followed by compensatory anti-inflammatory response syndrome (CARS) has gradually been discarded. Many studies suggest that, in the early stage of sepsis, concurrent inflammatory responses and immunosuppression can occur (Osuchowski et al., 2012; Hotchkiss et al., 2013). Nowadays, more patients survive the initial stage of sepsis owing to the development of early sepsis detection and treatment, though others have to stay in an intensive care unit for a long time, and experience a persistent inflammatory response, immunosuppression, malnutrition, muscle weakness characterized by myolysis, and sometimes death. Therefore, some researchers have put forward the concept of persistent inflammation, immunosuppression, and metabolic failure syndrome (PICS) (Gentile et al., 2012). According to this theory, after simultaneous inflammation and immunosuppression, the sepsis patient may recover immune homeostasis or develop an immunosuppression-induced secondary infection. The latter can result in a persistent inflammatory response and excessive energy consumption, causing further immune dysfunction and inflammatory responses (Delano and Ward, 2016; Mira et al., 2017; Nomellini et al., 2018).

Immunosuppression mainly manifests as decreased immune cell numbers and functions, including macrophage inactivation, decreased antigen presentation ability, and reduced lymphocyte proliferation (Delano and Ward, 2016). Additionally, inhibitory cytokine release is an important cause of immunosuppression (Kasten et al., 2010; Pillay et al., 2012). It is currently discovered that the organ dysfunction and high mortality rate among sepsis patients are mainly caused by the apoptosis of massive quantities of immune cells involved in the immune response (Fisher et al., 1996). Apoptosis is of great importance to immune homeostasis, but during sepsis, immune cell apoptosis can affect immune cell function and induce immune paralysis. In this situation, the risk of secondary infection significantly increases, and the mortality rate increases accordingly.

### Neutrophils

Neutrophils are important components of the innate immune system. They play a crucial role in suppressing and eradicating microorganisms and the survival of sepsis patients. They constitute the majority of cells in the bone marrow, and are the first immune cells to respond to foreign invaders in humans (Kolaczkowska and Kubes, 2013), representing important participants in the normal innate and adaptive immune responses.

After maturation in the bone marrow and entrance into the circulatory system, neutrophils can only survive for several hours. During this time, they clear extracellular pathogens mainly through direct phagocytosis and extracellular release of bactericidal substances (Davey et al., 2014). In recent years, research has indicated that neutrophils can also form neutrophil extracellular traps (NETs), which are primarily composed of networks of DNA from the neutrophils, to clear pathogens. NETs can restrict the supply of important nutrients required for pathogens, and they can physically restrain microorganisms. Moreover, histone and granulin in the NETs enable them to kill pathogens (Brinkmann et al., 2004).

In sepsis, the number, phenotype, and function of circulatory neutrophils are altered. In early sepsis, neutrophils can rapidly migrate from the bone marrow and, within several hours, the number of neutrophils can increase by 10-fold compared to under normal conditions (Manz and Boettcher, 2014). Additionally, the proportion of immature neutrophils in patients with septic shock increases (Demaret et al., 2015). These cells have a different phenotype and morphology from mature neutrophils, and much poorer functional performance (such as phagocytosis). Circulating immature neutrophils in patients with severe sepsis and septic shock have poor innate immunity performance (Drifte et al., 2013). Changes in the number and function of immature neutrophils increased the risk of death in septic shock patients (Demaret et al., 2015). Neutrophils had enhanced oxidative burst and phagocytosis functions, but their chemotaxis ability was greatly suppressed in sepsis (due to downregulation of integrin, selectin, and chemokine receptors, which altered their *in vitro* migration in relation to various chemical attractants) (Demaret et al., 2015). This reduced chemotaxis was even more obvious among patients who died of sepsis (Demaret et al., 2015), which is consistent with other research (Tavares-Murta et al., 2002). Additionally, an immunosuppressive neutrophil subset (CD16hiCD62low) was found in the blood of LPS-exposed subjects (Pillay et al., 2012). This subset might suppress T cell proliferation and function and might be involved in sepsis-related immunosuppression.

During sepsis, the cell membrane of neutrophils became stiffer and transformation-resistant, which was closely related to the severity of sepsis (Czaikoski et al., 2016). As a result, neutrophils accumulated in capillary beds, resulting in microvascular occlusion, especially in capillaries in the lungs and hepatic sinusoids (Czaikoski et al., 2016).

Delayed neutrophil apoptosis also occurs in sepsis, which may result in tissue injury and MODS (Hotchkiss and Nicholson, 2006). Inflammatory mediators such as granulocyte/macrophage colony-stimulating factor (GM-CSF) and IL-18 can regulate pro- and anti-apoptotic genes to regulate apoptosis (Tian et al., 2016). Notably, the upstream regulatory factors of these apoptotic mediators also participate in the delayed neutrophil apoptosis in sepsis.

During sepsis, neutrophils are recruited into the infection site as the first line of defense against bacterial and fungal pathogens. The classical white blood cell recruitment cascade includes retention, rolling, adhesion, crawling, and membrane penetration (Takatani et al., 2018). However, in sepsis, this reaction is disordered, accompanied by impaired recruitment of neutrophils into the infection site, and impaired neutrophil migration (Shen et al., 2017). This may be related to the downregulation of nitric oxide (NO) and inducible nitric oxide synthase (iNOS) (Shen et al., 2017), but the complete mechanism underlying the dysregulated neutrophil migration direction in sepsis remains unclear.

In sepsis, NETs serve as a double-edged sword, as they can trap pathogens but excessive NETs can cause organ injury and coagulation disorder (Czaikoski et al., 2016). Taken together, these phenotypic and functional changes of neutrophils in sepsis reduce the pathogen clearance rate, increase the risk of secondary (e.g., nosocomial) infection, and lead to poor prognosis.

### Mononuclear Macrophages

Mononuclear macrophages, including pre-monocytes in the bone marrow, monocytes in the peripheral blood, and fixed or free macrophages in tissues, are an important component of the innate immune system (Ardura et al., 2019). Typically, macrophages are derived from monocytes in the blood, whereas monocytes are derived from precursor cells in the bone marrow. Mononuclear macrophages are important cells in non-specific immune defense. They can non-specifically engulf and kill multiple pathogens. When pathogens invade the sterile environment of the body, mononuclear macrophages can recognize, engulf, and kill them in a process that can involve antigen presentation, and the mononuclear macrophages can also release inflammatory mediators to regulate the adaptive immune system (Hotchkiss et al., 2013). Most thymus-dependent antigens are engulfed and processed by macrophages, are used to form antigen peptide–major histocompatibility complex (MHC) complexes, and get expressed on the cell surface and presented to T cells. There are numerous adhesion molecules on the macrophage surface that can bind to the co-stimulatory receptors on T cells to produce a co-stimulatory signal, induce T cell activation, and initiate an immune response. Thus, mononuclear macrophages are not only a major factor in innate immunity, but they are also a bridge connecting innate and adaptive immunity. Sepsis is a complex syndrome involving both innate and adaptive immune responses, and it is of particular importance to explore the origin, differentiation, and function of macrophages during sepsis occurrence and development.

The most significant influence of sepsis on mononuclear macrophages is that it impairs their response to subsequent LPS stimulation or other inflammatory stimulation, which is called the endotoxin tolerance phenomenon (Venet and Monneret, 2018). Endotoxin-tolerant monocytes have weakened antigen presentation function and chemotaxis, but their killing and pathogen endocytosis abilities remain unchanged. In sepsis, mononuclear macrophages release fewer pro-inflammatory factors and more anti-inflammatory factors (Yang et al., 2013). This has been confirmed by analyzing the gene expression in monocytes from the blood of sepsis patients and macrophages from tissue, which exhibit upregulation of anti-inflammatory genes and downregulation of pro-inflammatory genes (Monneret et al., 2004; Cavaillon and Adib-Conquy, 2006; Biswas and Lopez-Collazo, 2009).

Human leukocyte antigen-DR (HLA-DR) on the monocyte surface is the key molecule involved in foreign antigen processing and presentation. When pathogenic microorganisms invade the body, the body immediately initiates an immune response, and then monocytes and other APCs present antigen components to specific lymphocytes with the assistance of HLA-DR. During sepsis, monocytes downregulate surface HLA-DR, indicating immune paralysis in relation to monocytes (Cazalis et al., 2013). Monocytes with HLA-DR downregulation exhibit impaired pro-inflammatory cytokine release, antigen presentation, and induction of T cell proliferation (Wolk et al., 2000). HLA-DR expression on the monocyte surface is of great value in predicting the prognosis of sepsis patients with immuno-inflammatory dysfunction (Volk et al., 1996; Lekkou et al., 2004).

During sepsis, macrophages can differentiate into M2 macrophages. These differentiated macrophages produce arginase-1, which converts arginine into urea, thus suppressing iNOS-induced NO production from arginine and thereby decreasing the killing of pathogens. Therefore, in the late stage of sepsis, the body has a weakened ability to resist pathogens; in other words, the occurrence of immunosuppression may be related to the M2 differentiation of macrophages (Lawrence and Natoli, 2011).

### DCs

First discovered by the Canadian researcher Steinman in 1973, DCs are the most potent APCs. They are named because they have numerous dendrite- or pseudopodium-like protrusions when they mature. They highly express cell-surface MHCII molecules. They are derived from bone marrow multipotential hematopoietic stem cells, and they are highly heterogeneous and extensively distributed at sites such as the skin, airways, and lymphoid organs. DCs are the most potent specialized APCs in the body, and they effectively absorb, process, and present antigens. Immature DCs show strong migration ability, whereas mature DCs can effectively activate primary T cells and play a central role in initiating, regulating, and maintaining the immune response. DCs can migrate to lymphoid organs, stimulate primary T cell proliferation, and exhibit relatively specific surface markers. Therefore, DCs, which are extensively distributed in lymphoid and non-lymphoid tissues, are considered the initiators of the immune response and maintain homeostasis in the body (Heath and Carbone, 2001; Steinman and Banchereau, 2007). Generally, DCs can be classified as classical DCs (cDCs) and plasmacytoid DCs (pDCs). The former are mainly derived from myeloid progenitor cells, and they mainly express CD11c and MHCII on their surface. Once tissue injury or pathogen invasion is detected, cDCs upregulate the surface co-stimulatory molecules CD80 and CD86, secrete the pro-inflammatory cytokines IL-6 and TNF-α, and rapidly migrate to lymph node T cell zones to initiate a T cell-mediated adaptive immune response. In contrast, pDCs represent the major source of type I IFN (Geissmann et al., 2010; Bouras et al., 2018).

The reduced DCs number and dysfunction are key causes underlying sepsis-related immunosuppression and secondary infection or even death. Splenic and circulatory DC counts significantly decrease in sepsis patients, with both pDCs and cDCs being affected (Guisset et al., 2007; Grimaldi et al., 2011; Dreschler et al., 2012). Moreover, the number of circulatory DCs was markedly decreased in sepsis patients who died compared to those who survived (Guisset et al., 2007; Grimaldi et al., 2011). In a mouse model of cecal ligation puncture-induced sepsis, the total splenic cell count decreased by 50% at 2 days after sepsis, while CD11c + cDC and CD11c-B220 + CD19^−^pDC counts decreased by 75 and 50%, respectively, compared to the counts in the control group. The reduced DC count directly causes insufficient CD8^+^ T cell activity (Strother et al., 2016). The reduced DC count is currently thought to be induced by enhanced sepsis-related apoptosis. Importantly, the changes in DC count and function last for several weeks after hospital admission (Wen et al., 2008), and it takes several months for them to return to normal levels after sepsis.

As mentioned, during sepsis, DC functions are also changed. In the remaining circulatory DCs in sepsis patients, HLA-DR, CD80, and CD86 are downregulated, while IL-10 production is increased (Faivre et al., 2012), which is consistent with their reduced ability to induce effector T cell responses and their ability to prevent the response of T cells and the proliferation of Treg cells (Faivre et al., 2007; Wen et al., 2008). These DCs express a low level of IFN regulatory factor 4 (IRF4), which is directly related to their antigen presentation ability (Roquilly et al., 2017). Additionally, during sepsis, DCs produce lower levels of pro-inflammatory cytokines (such as IL-12 and TNF-α) and higher levels of anti-inflammatory cytokines (such as IL-10 and TGF-β) (Wen et al., 2008; Faivre et al., 2012; Roquilly et al., 2017). Normally, DCs activated at 6–24 h after bacterial infection can induce neutrophil, NK cell, and mononuclear macrophage immune responses. However, during sepsis, DCs undergoing immune paralysis may lead to decreased scavenging of bacteria by innate immune cells, including NK cells. Much data suggests that the mechanisms underlying DC dysfunction include apoptosis induction, Wnt signaling pathway activation, reactive oxygen species (ROS) production, TLR-dependent signaling, and abnormal epigenetic regulation (Wu et al., 2017).

### NK Cells

NK cells were discovered in humans and mice in the 1970s. They are innate immune cells that are also called congenital lymphocytes. Unlike T and B cells, NK cells are lymphocytes that can non-specifically kill tumor cells and virus-infected cells with no need for sensitization in advance. The exact origin of NK cells remains unclear, though they are generally considered to be directly derived from the bone marrow, and their differentiation and development are dependent on the bone marrow and thymus microenvironments. NK cells are the key effector cells in the innate immune system. They are similar to large granular lymphocytes in terms of their morphology, and their volume is twice that of red blood cells. They are extensively distributed in diverse tissues, mainly the abdominal cavity, placenta, and uterine mucosa, and especially the liver. CD3^−^CD56^+^ cells are generally considered NK cells, which account for about 5–20% of monocytes in the blood (Cossarizza et al., 2017). NK cells can produce rapid and non-specific innate immune responses to cancer cells and cells infected with intracellular pathogens (Campbell and Hasegawa, 2013; Padro and Luong, 2016). Moreover, NK cells play important roles in initiating the host defense and regulating the innate and adaptive immune responses. In addition to cytotoxic effects, NK cells can secrete pro-inflammatory cytokines, such as TNF-α and IFN-γ, which can enhance the pro-inflammatory and anti-microbial functions of other white blood cell populations (Guo et al., 2018). Based on the cell-surface expression of CD56 and CD16, NK cells can be divided into CD56dimCD16bright and CD56brightCD16−/dim NK cell subsets. Typically, the former possesses stronger cytotoxicity and expresses higher levels of killing immunoglobulin receptor (KIR), whereas the latter exhibits improved proliferation and IFN-γ and TNF-α secretion in response to pro-inflammatory cytokine stimulation (Kumar, 2019).

Many studies indicate that the number of NK cells decreases during sepsis, which is related to poor prognosis, including death. One study reported that the NK cell count decreased in the blood of sepsis patients within 24 h of onset (Boomer et al., 2012). Another study reported that the number of NK cells in sepsis patients continuously decreased in the first 14 days after hospitalization, and the NK cell number was markedly decreased in Gram-negative sepsis patients compared to Gram-positive patients (Holub et al., 2000). The number of NK cells may be related to the increased apoptosis and the migration of NK cells from the peripheral blood to the infection site during infection.

NK cells also show dysfunction during sepsis. NK cell functions, such as cytotoxicity and cytokine secretion (IFN-γ), significantly decrease during sepsis in mice and patients (Forel et al., 2012). Similarly, the expression of surface receptors expressed on immune competent cells, such as NKG2D are lower in sepsis patients than non-sepsis patients, which may reduce NK cell cytotoxicity (Kjaergaard et al., 2015). This persistent NK cell dysfunction may be closely associated with sepsis-induced immunosuppression. Persistent NK cell depletion and dysfunction may impair the host defense against pathogens. As a result, sepsis patients are more susceptible to secondary infection and viral reactivation.

Although NK cells are of great significance in early infection control, their excessive response amplifies the inflammation and results in organ and tissue damage. Some studies suggest that, during infection or endotoxin attack, NK cells represent the promoter of systemic inflammation. In the process of sepsis, NK cells may be excessively activated and produce excessive amounts of IFN-γ and TNF-α, thus leading to systemic inflammation aggravation, MODS, and an increased risk of mortality (Sherwood et al., 2003; Sherwood et al., 2004; Tao and Sherwood, 2004). Suppressing NK cell function by knocking out *IL-15* significantly improved the survival rate of septic mice (Guo et al., 2017). In addition, sepsis patients with a reduced number of NK cells have a higher survival rate (de Pablo et al., 2012). These results reveal that the excessive activation of NK cells results in poor prognosis of sepsis patients. The adverse effects of NK cells are mediated by their ability to amplify the pro-inflammatory response or directly cause organ injury, possibly *via* cytotoxicity.

### Lymphocytes

When the innate immune system is insufficient to defend against pathogens, the adaptive immune response, dominated by T and B cells, is of particular importance. B cells mainly mediate humoral immunity. They can produce antigen-specific antibodies with the assistance of T cells. These antibodies can neutralize toxins, activate the complement system, and facilitate phagocytosis of pathogens by mononuclear macrophages. T cells mainly mediate cellular immunity, so they play an important role in killing various pathogens. T cells are mainly derived from bone marrow lymphoid stem cells. After differentiation, growth, and maturation in the thymus, they are distributed to the systemic immune organs and tissues *via* the lymphatic and blood circulation to exert immune functions. Using cell-surface T cell receptors, T cells can recognize microbial peptides bound to MHC molecules on APCs, including mononuclear macrophages and DCs, thus inducing a primary immune response. Pluripotent stem cells can transform into lymphoid progenitor cells and migrate to the thymus. Thereafter, thymosin induces them to undergo a series of orderly differentiation processes and gradually form a T cell library that can recognize diverse antigens.

T cells can be divided into “helper” CD4^+^ T cells and “killer” CD8^+^ T cells, based on their different growth paths and biological functions. Naive CD4^+^ T cells released from the thymus further differentiate into effector T cells and Treg cells. Depending on the different cytokines secreted, effector T cells can be classified into Th1, Th2, and Th17 cells. Th1 and Th17 cells produce pro-inflammatory mediators, whereas Th2 cells generate anti-inflammatory factors. Moreover, T cells can exhibit an immune memory phenotype (Xie et al., 2019).

The lymphocyte count in the circulation significantly decreases within 24 h after the diagnosis of sepsis. Based on the autopsy results of sepsis patients, large quantities of immune cells in the spleen of patients dying of sepsis underwent apoptosis, including CD4^+^ T cells, CD8^+^ T cells, B cells, and DCs (Boomer et al., 2011), which is an important cause of sepsis-related immune paralysis. Moreover, compared to naive T cells, memory T cells are more susceptible to apoptosis during sepsis. In septic mice, the numbers of memory CD4^+^ and CD8^+^ T cells in the spleen significantly decreased (Xie et al., 2019). The T cell apoptosis mechanisms include upregulation of pro-apoptotic protein Bim and downregulation of anti-apoptotic protein Bcl-2 (Weber et al., 2008), and Bcl-2 and Bcl-xL upregulation improves the prognosis in animal models of sepsis (Schwulst et al., 2008). Additionally, extracellular HMGB1 induces T cell apoptosis *via* the intrinsic apoptosis pathway, while upregulation of Mitofusin 2 (Mfn2) protects T cells (Zhao et al., 2012; Wu et al., 2014).

The non-apoptotic CD4^+^ and CD8^+^ T cells in sepsis cases display inactivity or failure, with large phenotypic and functional impairments. Clinical research has shown that inhibitory molecules are upregulated in T cells in sepsis patients, and T cells have reduced secretion of cytokines. Further, autopsy results suggest that CD69, PD-1, and CD25 on T cells from the spleen of patients dying of sepsis were upregulated, whereas CD127 and CD28 were downregulated (Boomer et al., 2011). Further research indicated significant upregulation of co-inhibitory receptors, such as PD-1, cytotoxic T lymphocyte-associated antigen-4 (CTLA-4), B and T lymphocyte attenuator (BTLA), T cell immunoglobulin mucin03 (TIM-3), and lymphocyte activation gene-3 (LAG-3), on T cells from the blood of sepsis patients (Boomer et al., 2012). Epigenetic reprogramming also participates in T cell dysfunction during sepsis. In septic mice, histone methylation and chromatin remodeling are observed in the promoters of genes that encode adhesive protein Annexin-A1 (ANXA1) and GATA-binding protein 3 (GATA-3), leading to lymphocytic incompetence (Huang et al., 2016). The upregulation of T cell co-inhibitory receptors, the reduced secretion of cytokines, and epigenetic reprogramming jointly result in T cell exhaustion, which is another important mechanism underlying immune paralysis in the late stage of sepsis.

Another type of immune cell in sepsis deserving our attention is Treg cells. These cells can secrete the anti-inflammatory factor IL-10 and suppress the excessive inflammatory response in early sepsis (Mosser and Zhang, 2008). However, some researchers believe that in the immune paralysis stage of sepsis, the increased Treg cell proportion and their inhibitory function may aggravate immunosuppression in sepsis, leading to PICS (Tatura et al., 2015). Despite this, one study showed that the survival rate of septic mice did not increase after Treg cell depletion using anti-CD25 antibody (Hein et al., 2010). Likewise, Increased natural CD4^+^CD25^+^ regulatory T cells and their suppressor activity did not affect the mortality of septic mice (Scumpia et al., 2006). Moreover, at 24 h after sepsis, both Treg cell-deficient and wildtype mice exhibited a strong inflammatory response, along with immune cell migration toward the peritoneum and bacterial seeding (Kühlhorn et al., 2013). However, in the late stage of sepsis, the wildtype mice rapidly recovered from sepsis compared to the Treg cell-deficient mice (Kühlhorn et al., 2013). Therefore, although increasing the Treg cell proportion in early sepsis may be beneficial to suppress the inflammatory response, excessive Treg cell suppression in the late stage appears to be detrimental; nonetheless, this hypothesis requires more evidence from basic and clinical research.

## Pathophysiological Mechanisms of Sepsis

### Imbalance Between SIRS and CARS

As a result of the in-depth understanding of sepsis, SIRS and CARS are now known to be able to occur at the same time in the early stage (László et al., 2015; Taeb et al., 2017). The combined effect of the two states can cause tissue damage. SIRS is predominant in the early stage, which can lead to symptoms such as high fever, tachypnea, hypotension, and tachycardia (van der et al., 2017). During this stage, M1 macrophages release excessive pro-inflammatory cytokines, such as IL family members (IL-1, IL-3, IL-6, IL-8 etc.), TNF-α, and IFN-γ, which aggravate the immune damage (Liu et al., 2014). In most cases, the innate immune response can eliminate the invading pathogens. However, when the pathogens dominate, the host response may become unbalanced.

With the progress of sepsis, the inflammatory reaction gradually changes from overactivation to immunosuppression, with the main manifestations being decreased immune cell counts and dysfunction. As the number of immature neutrophils increases, their ability to engulf pathogens decreases, which seriously affects the removal of pathogens and the body’s resistance to pathogens (McDonald, 2018). M2 macrophages secrete high levels of anti-inflammatory cytokines, such as IL-4 and IL-10, thereby reducing the immune function (Liu et al., 2014; Chen et al., 2019). During this stage, regarding the adaptive immune system, T cell apoptosis increases, Treg cell proportionincreases, and a Th1/Th2 imbalance occurs (Luan et al., 2015; Yoon et al., 2017). Under the combined action of the abovementioned mechanisms, the body loses its normal immune function and gradually enters the phase of immune paralysis or immunosuppression. DAMPs can be recognized by and activate the PRRs of many PAMPs, leading to vicious cycles of persistent immune activation and organ dysfunction (Chan et al., 2012; Deutschman and Tracey, 2014). With the advancement of monitoring and treatment technologies, some sepsis patients can survive the acute phase but develop the chronic critical illness PICS.

### Immune Dysfunction and Autophagy

Autophagy refers to the orderly spontaneous death of cells after stimulation in order to maintain homeostasis of the internal environment, involving multiple proteins and organelles. It is also involved in the interactions between the immune system and pathogens. The extremely complex pathophysiological process of sepsis involves not only an inflammatory reaction imbalance and immune dysfunction but also the dysregulation of autophagy. The occurrence and development of abnormal autophagy are closely related to MODS in sepsis patients.

Autophagy is activated in the early stage of sepsis and then enters a restricted phase. The functions of autophagy are to protect sepsis patients from developing MODS by preventing apoptosis, maintaining the balance between pro-inflammatory and anti-inflammatory cytokine production, and ensuring optimal mitochondrial function. When these processes are out of balance, sepsis and organ dysfunction occur (Ho et al., 2016). Autophagy can be triggered by inhibiting nicotinamide phosphoribosyltransferase (NAMPT) and reducing the secretion of pro-inflammatory cytokines such as TNF-α, IL-1β, and IL-6 (Li et al., 2018). Macrophages with autophagy-related 16-like 1 (*Atg16L1*) deficiency oversecrete IL-1β and IL-18 under LPS stimulation (Saitoh et al., 2008). Additionally, autophagy-deficient monocytes without *Atg7* exhibit mitochondrial dysfunction, resulting in excessive secretion of IL-1β (van der et al., 2014). Furthermore, deletion of the key autophagy gene light chain 3 (*LC3)* in two mouse models of sepsis (cecal ligation puncture and intraperitoneal LPS injection) exhibited significantly increased IL-1β and IL-18 secretion (Nakahira et al., 2011). This suggests that autophagy deficiency participates in sepsis by leading to an increase in the release of inflammatory factors. The reduction in autophagy promotes the inflammatory reaction and leads to cell death, which further aggravates sepsis (Zhang et al., 2018).

Autophagy in the innate immune system plays important protective roles in infectious, autoimmune, and inflammatory diseases. In sepsis, macrophages express surface receptors, identify pathogens, and release a large quantity of pro-inflammatory factors, thereby enhancing autophagy, which in turn negatively regulates macrophages. Macrophage polarization decreases inflammasome activation and inflammatory factor release, thereby exerting a protective effect. Autophagy inhibition or even lack of macrophages was observed in the experiment. The number of damaged mitochondria increased, producing an excessive amount of ROS. The number of damaged phagosomes containing bacterial components also increased. These increases activated NOD-like receptor family pyrin domain-containing 3 protein (NLRP3), which induced an inflammatory reaction. Increased autophagy can reduce the inflammatory reaction mediated by this pathway, protecting the organism (Qiu et al., 2019).

As a type of white blood cell, neutrophils play an important role in the immune response. Research using phorbol 12-myristic 13-acetate (PMA) to stimulate neutrophils obtained from patients with early-stage sepsis showed that promoting autophagy increased NET formation. The protective functions of NETs include removing microorganisms and participating in the inflammatory reaction (Park et al., 2017).

In addition to affecting innate immune cells, autophagy’s protective effect also includes adaptive immune cells, involving a variety of cellular receptors and intracellular signaling pathways. Autophagy can maintain homeostasis by regulating T cells. For example, CD4^+^ T cells can undergo autophagy in response to environmental changes to alter their biological functions (Jacquin and Apetoh, 2018). In a mouse model of cecal ligation puncture-induced sepsis with T cell-specific deletion of a mouse-specific autophagy gene (*Atg7* or *Atg5*), peripheral CD4^+^ and CD8^+^ T cells rapidly underwent apoptosis and the number of secondary lymphoid organs decreased (Oami et al., 2017). Even with antigen stimulation, the T cells were unable to proliferate (Oami et al., 2017). In this model, reduced autophagy inhibited T cell activation, ultimately increasing the bacterial load (Oami et al., 2017). The increased mortality of the mice shows that autophagy-deficient T cells can cause immune dysfunction in sepsis (Oami et al., 2017).

Autophagy has dual effects in the body. Under mild stimulation, autophagy promotes cell survival by acting as a quality control mechanism. Under severe or chronic stimulation, excessive or insufficient autophagy can cause excessive self-degradation and accumulation of toxic substances, which can lead to cell death. Therefore, treating sepsis-related organ injury by regulating autophagy may become an effective sepsis treatment in the future. Autophagy regulation differs between different organs, which exhibit different physiological levels of autophagy and functions related to autophagy. Current studies on autophagy are still in the basic research stage, and the regulatory mechanisms at the cellular level need to be identified. Furthermore, the mechanisms underlying the transformation of autophagy from being protective to promoting cell death remain unclear, and further exploration is required using both animal experiments and clinical trials.

### Gene Polymorphisms

Gene polymorphism refers to the variation of gene sequences in the same population. Gene polymorphisms determine the susceptibility or tolerance of the body to stress stimuli and the diversity of clinical manifestations and drug therapeutic effects, thus further affecting an infected host’s gene expression and prognosis (Behnes et al., 2013; Thompson et al., 2014). With the deepening understanding of genomics, genetic differences are considered to be the internal basis for the occurrence and development of many diseases. Sepsis is caused by the joint action of environmental and genetic factors, and its occurrence and development can be independently or synergistically affected by various genetic variations.

The mechanisms underlying the effects of gene polymorphisms on sepsis have not yet been clarified. Current research on sepsis-related gene polymorphisms mainly involve TNF family members, IL family members, heat shock protein 70 (HSP70) (Giacconi et al., 2014), CD14, plasminogen activator inhibitor (PAI-1), and mannose-binding lectin (MBL) (Özkan et al., 2012; Mao et al., 2017). Polymorphisms of these genes are closely associated with 28-days mortality among sepsis patients (Mansur et al., 2015; Giamarellos-Bourboulis and Opal, 2016). Studies on the relationships between gene polymorphisms and sepsis can not only reveal the pathogenesis of sepsis at the gene level, but also provide a new theoretical basis for the early identification of sepsis and MODS, gene therapy, and prognostic prediction. Genetic studies have shown that host genetic variants can be used as biomarkers of sepsis susceptibility. In addition, recent research has shown that rare harmful gene variants can predict the post-sepsis course and some may even have protective effects (Taudien et al., 2016). Haplotype variations related to TNF-α can prevent patients with SIRS from developing sepsis (Retsas et al., 2018). However, these variations have no effect on disease severity or mortality, so the associations between gene polymorphisms and sepsis prognosis need further study.

### MODS

It is rare that sepsis causes dysfunction of a single organ, as it can affect almost all organs of the body. In clinical practice, the six commonly evaluated organ systems are the cardiovascular, respiratory, renal, nervous, blood, and liver systems. The sequence usually involves dysfunction of the respiratory and cardiovascular systems, impairment of liver and kidney function and blood coagulation, followed by disorders of the gastrointestinal and central nervous systems. As the number of failing organs increases, the mortality rate increases (Vincent et al., 2006; Sakr et al., 2012). Sequential Organ Failure Assessment (SOFA) is used to objectively quantify organ dysfunction, and it is a valuable indicator of prognosis (Vincent et al., 1996).

Patients with sepsis usually suffer from coagulopathy, which often causes thrombocytopenia and even disseminated intravascular coagulation (DIC) in the late stage of the disease, leading to significant deterioration. This is mainly due to the activation of the coagulation pathway, the inhibition of the anticoagulation pathway, and the reduced fibrinolytic system function (Tsao et al., 2015; Lipinska-Gediga, 2016). Endothelial cells play an important role in regulating the vasomotor tone, movement of cells in and out of tissues, blood coagulation system, and balance of inflammation and anti-inflammatory signals. In sepsis, endothelial cell dysfunction can cause extensive tissue edema, which further aggravates the condition (Aird, 2003). In severe sepsis, changes in endothelial cells are closely related to changes in the barrier function of many organs. The dysfunction of the alveolar epithelial barrier and pulmonary interstitial and alveolar edema can cause acute respiratory distress syndrome (Matthay et al., 2012). The combined destruction of the endothelial and epithelial barriers is the major mechanism underlying extensive organ dysfunction. This can cause bacterial translocation due to intestinal dysfunction and intestinal injury triggered by digestive fluids. Acute kidney injury is also common in severe sepsis (Alobaidi et al., 2015). The mortality of sepsis patients with acute kidney injury or DIC was 2–3 times higher than that of patients with sepsis only (Kudo et al., 2018).

The prevention and treatment of MODS in sepsis mainly involves early monitoring and identification, drug treatments, and organ function support, such as mechanical ventilation, blood purification, and extracorporeal membrane oxygenation (ECMO).

## Immunotherapy for Sepsis

The process of understanding sepsis has been very complex, and it was previously suggested that sepsis mainly occurs due to an excessive inflammatory immune response of the host to infection. Consequently, in the 1990s, many clinical studies focused on restricting excessive inflammation, but substantial success was not achieved. These disappointing results, together with the advances in the understanding of the pathophysiology of sepsis, have allowed sepsis to be recognized as a dysregulated inflammatory response, rather than excessive inflammation. Restoring immune homeostasis may be beneficial for sepsis patients. Constructing and optimizing a dynamic immune system treatment regimen based on anti-infection treatment, fluid replacement, organ function support, and timely use of immunomodulatory interventions may improve the prognosis of sepsis patients.

### Drugs to Reduce the Cytokine Storm

Regarding the sepsis-related cytokine storm, the cytokines that induce excessive pathological inflammation remain unclear. Thus, antibiotics remain the major means of treatment. Currently, clinical trials have not shown cytokine-specific monoclonal antibodies to be effective. However, research is being conducted on glucocorticoids, cytokine antagonists, ulinastatin, and blood purification to reduce the cytokine storm.

#### Glucocorticoids

Glucocorticoids have been used for a few decades to treat sepsis, and their advantages and disadvantages remain disputed. Their major function is to downregulate genes regulating the inflammatory response (including NF-kB and AP-1) to suppress innate immunity (Heming et al., 2018). However, some key mechanisms of action remain unclear. Hydrocortisone can reduce the serum levels of pro-inflammatory mediators (TNF, IL-1, IL-6, and IL-8) in patients with septic shock, while inhibiting the activation of endothelial cells (based on the level of soluble E-selectin) and neutrophils (Keh et al., 2003; Oppert et al., 2005). A recent multicenter, double-blind, factorial randomized controlled trial (RCT) suggested that hydrocortisone combined with fludrocortisone compared to placebo improved the 90-days mortality rate of sepsis patients (Annane et al., 2018). However, no clinical trial has verified that hydrocortisone monotherapy can improve the survival rate of septic shock patients (Heming et al., 2018). The 2018 *Guidelines for Emergency Treatment of Sepsis and Septic Shock in China* state that intravenous injection of 200 mg hydrocortisone can be used in patients with unstable hemodynamics after the use of vasoactive drugs and fluid replacement (Cao et al., 2018). Hopefully, multicenter RCTs will be carried out to determine the safety and effectiveness of glucocorticoids for treating sepsis.

#### Cytokine Antagonists

The pathogenic process of sepsis is accompanied by the production of excessive cytokines to regulate the immune–inflammatory response of the body. Therefore, theoretically speaking, it seems to be a promising strategy to modulate these cytokines to reduce the disadvantages of the sepsis-related host response. However, in the 1990s, the use of the fusion proteins TNF receptor–Fc and p55 TNF receptor–IgG to antagonize TNF was not shown to reduce the mortality rate of septic shock patients (Fisher et al., 1996; Abraham et al., 1997). In another study, when the serum IL-6 level in patients was >1,000 pg/ml, the 28-days mortality rate significantly decreased when the neutralizing anti-TNF-α antibody afelimomab was used to adjust IL-6 to appropriate levels (Panacek et al., 2004). However, patients with a low IL-6 level did not benefit from this treatment (Panacek et al., 2004). In the treatment of critical COVID-19 patients, the anti-IL-6 receptor (IL-6R) antibody tocilizumab, which can bind to both membrane-bound and soluble IL-6R, blocked downstream signal transduction and improved the prognosis (Xu et al., 2020). In another two studies, anti-IL-6 antibody combined with corticosteroids was more beneficial than the monotherapies (Remap-Cap et al., 2021; RECOVERY Collaborative Group, 2021).

Although many experiments involving mice with sepsis suggest that the use of antibodies against pro-inflammatory cytokines can antagonize their activities, alleviate the inflammatory response, and thus improve the survival rate, these therapies have not achieved satisfactory effects in sepsis patients (Fisher et al., 1996).We believe that if administered at an appropriate time point, cytokine regulation is definitely beneficial for some patients. However, if the inflammatory cascade has already passed the irreversible point, anti-cytokine treatment may not provide more benefits. Additionally, it is inadvisable to block some cytokines at the early stage of the disease. This is because many cytokines are major regulators of inflammation and immunity, and anti-cytokine treatment at the early stage of infection may block beneficial immune responses. Consequently, we believe that the timing, dosage, and target cytokine levels are the keys to successful therapeutic effects of anti-cytokine treatment in sepsis patients.

#### Ulinastatin

Ulinastatin is a natural anti-inflammatory substance found *in vivo*. It suppresses the production and release of inflammatory mediators to protect the vascular endothelium, and it can be used to treat sepsis-related acute circulatory failure. Ulinastatin can reduce the pro-inflammatory levels (including TNF-α, IL-6, and IFN-γ), increase the anti-inflammatory factor IL-10 level (Tao et al., 2017), and promote the balance between pro-inflammatory and anti-inflammatory responses, thus blocking the cytokine storm induced by the vicious circle of inflammatory responses. Animal studies suggest that high-dose ulinastatin achieves a comparable anti-inflammatory effect to glucocorticoids (Xu et al., 2018). In 2017, a meta-analysis of eight RCTs suggested that ulinastatin combined with thymosin α1 (Tα1) in sepsis patients suppressed pro-inflammatory factor production, reduced the Acute Physiology and Chronic Health Evaluation (APACHE) II score, shortened the durations of mechanical ventilation, and improved the 28-days survival rate (Liu et al., 2017). In addition, it does not induce immunosuppression and causes fewer side effects than glucocorticoids.

#### Blood Purification

Continuous blood purification (CBP) has become an important means of life support treatment for critically ill patients. In patients treated with CBP, pro- and anti-inflammatory responses remain at low levels, which can block the cytokine storm at the early stage of sepsis, thus blocking the development of life-threatening sepsis (Bagshaw et al., 2008). Recent studies have focused on the use of plasma exchange for treating sepsis. A clinical study reported that therapeutic plasma exchange significantly reduced the pro-inflammatory cytokine levels and improved the hemodynamics of septic shock patients (Knaup et al., 2018). The blood purification techniques applied in the clinic can rapidly scavenge cytokines and inflammatory mediators in the body. However, relevant high-quality RCTs are lacking, and no treatments of this kind are currently recommended in sepsis treatment guidelines (Ronco et al., 2003).

### Drugs to Enhance Innate Immunity

#### IFN-γ

IFN-γ is mainly produced by Th1 and NK cells, and it is the marker cytokine of Th1 cells. It can enhance the bacterial phagocytosis function of macrophages, promote scavenging of bacteria, and upregulate PRRs to accelerate antigen presentation by APCs (Burke and Young, 2019). In 1997, Döcke et al. (1997) first verified that IFN-γ treatment significantly upregulated mHLA-DR, enhanced TNF-α production by mononuclear cells. IFN-γ secretion after *in vitro* stimulation was impaired in peripheral blood mononuclear cells (PBMCs) from sepsis patients compared to those from healthy controls (Boomer et al., 2012). Moreover, IFN-γ secretion was decreased in PBMCs from sepsis patients who died compared to those from sepsis patients who survived. Regardless of these promising preliminary clinical results, special attention should be paid to the clinical safety of IFN-γ, as it is a pro-inflammatory cytokine.

#### GM-CSF

GM-CSF is a hematopoietic growth factor that stimulates the proliferation and differentiation of multiple immune cells from myeloid stem cells to mature granulocytes. During sepsis, GM-CSF can enhance the phagocytosis and antimicrobial activity of neutrophils and mononuclear macrophages to improve immunity (Borriello et al., 2019). In 2002, a randomized, double-blind, placebo-controlled phase II study showed that intravenous injection of low-dose GM-CSF (3 μg kg^−1^·d^−1^) improved the oxygenation index in sepsis patients with respiratory insufficiency, but it did not improve the 30-days survival rate (Presneill et al., 2002). In 2006, a double-blind, placebo-controlled RCT showed that low-dose GM-CSF (3 μg kg^−1^·d^−1^) reduced the antibacterial treatment duration, length of hospital stay, and infection-related complications in sepsis patients with abdominal infection (Orozco et al., 2006). These two clinical trials did not reduce the in-hospital mortality of sepsis patients. However, in sepsis patients, GM-CSF restored the HLA expression on mononuclear cells, and increased TNF release by white blood cells after LPS stimulation (Nierhaus et al., 2003). In 2009, a double-blind RCT showed that GM-CSF (4 μg·kg^−1^·d^−1^) significantly upregulated mHLA-DR and significantly reduced the durations of ventilator use, hospital stay, and intensive care unit stay, Compared with the control group of patients (Meisel et al., 2009). In 2018, a clinical trial of 10 patients treated with GM-CSF (3 μg·kg^−1^·d^−1^ on four consecutive days) showed that phagocytosis by neutrophils increased by 50%, which was significantly higher than the rate in the placebo group (Pinder et al., 2018). The study showed that GM-CSF may improved phagocytosis by innate immune cells, reduced the incidence of secondary infections, and thus improved the prognosis of sepsis patients. However, the optimum dose and treatment duration should be further explored.

### Drugs to Enhance Adaptive Immunity

#### IL-7

IL-7 is produced in the bone marrow and thymus, and it is necessary for the maturation and survival of T cells. In septic mice, IL-7 reduced lymphocyte apoptosis, induced T cell proliferation, promoted the migration of white blood cells to the infection site, and improved the survival rate (Shindo et al., 2017). Recently, a prospective, randomized, double-blind,placebo-controlled phase II RCT on the therapeutic effect of recombinant human IL-7 (CYT107) in 27 septic shock patients with lymphopenia showed that the absolute lymphocyte count and CD4^+^ and CD8^+^ T cell counts significantly increased (Francois et al., 2018). Moreover, T cells maintained favorable activation, and CYT107 did not induce an excessive inflammatory response or aggravate organ dysfunction. This trial was the first on immunoadjuvant therapy in immunodeficient sepsis patients, and the results indicated the relative safety of IL-7. This treatment represents a potential new approach for treating sepsis patients by recovering adaptive immunity.

#### Immunoglobulin (Ig)

Ig is a natural protein secreted by B cells. It can be used to neutralize toxins in the body, reduce immune cell apoptosis, suppress inflammation, and mediate phagocytosis by macrophages. Therefore, supplementing with Ig may improve the prognosis of sepsis patients. Clinical studies on the effect of intravenous immunoglobulin (IVIg) in patients with sepsis (Werdan et al., 2007) and patients with severe SIRS after cardiac surgery (Werdan et al., 2008) showed that it did not reduce the mortality rate. RCTs obtained inconsistent results, and a meta-analysis of these studies did not indicate overall benefits. Therefore, IVIg was not recommended in the guidelines of the most recent Surviving Sepsis Campaign in 2016 (Rhodes et al., 2017). However, a meta-analysis in 2019 (19 studies with >150 patients) suggested that intravenous immunoglobulin rich in IgM (IVIgM) reduced the risk of death among sepsis patients (Kalvelage et al., 2019). This suggests that IgM-rich preparations might help to kill bacteria in the body, thus improving the prognosis of sepsis patients. The routine use of Ig for treating sepsis is not currently recommended, but Ig can be considered in sepsis patients with low Ig.

#### Thymosin α1 (Tα1)

Tα1 is an endogenous peptide secreted by organs such as the thymus. It is a natural small molecule that has important regulatory function in innate and adaptive immunity. It can activate DCs, improve NK cell activity, directly enhance macrophage-mediated phagocytosis and antibacterial effects, upregulate HLA-DR and programmed death ligand 1 (PD-L1) on mononuclear cells, increase the T cell count and activity, and enhance the antibacterial activity of Th1 cells (Camerini and Garaci, 2015; Van der et al., 2017). In recent years, a series of clinical trials on Tα1 for sepsis have been conducted in China. In 2013, a multicenter, single-blind RCT in China suggested that the 28-days mortality rate in the Tα1 group significantly decreased compared to that in the placebo group (26 vs. 35%, relative risk = 0.74), HLA-DR was significantly upregulated, and there were no severe adverse drug reactions (Wu et al., 2013). Therefore, the use of Tα1 may improve the prognosis of severe sepsis patients. In a recent retrospective cohort study of 334 critical COVID-19 patients at eight centers, Tα1 significantly increased the 28-days survival rate and improved the oxygenation index (Wu et al., 2020). A meta-analysis of 19 studies reported that Tα1 improves the prognosis of sepsis patients; unfortunately, the overall sample size was small. Therefore, we still need large high-quality RCTs to further verify the role of Tα1, optimal dose, treatment duration, and target population among sepsis patients.

#### Anti-PD-L1 Antibody/anti-PD-1 Antibody

Anti-Programmed death-ligand 1 (PD-L1) antibody/anti-programmed cell death -1 (PD-1) antibody can restore T cell function by blocking PD-1/PD-L1 signaling, and it is a novel anti-tumor immunotherapy regimen. Sepsis and cancer share many similarities regarding immune mechanisms. PD-1 and PD-L1 were significantly upregulated in mononuclear cells and CD4^+^ cells from septic shock patients compared to healthy subjects (Guignant et al., 2011; Shao et al., 2016). This upregulation was closely related to the high nosocomial infection and mortality rates. In mice with sepsis, this treatment suppressed apoptosis, reversed immune dysfunction, and improved the survival rate (Brahmamdam et al., 2010). Recently, a phase I RCT (NCT02576457, BMS-936559) of 24 patients with sepsis-related immunosuppression verified the safety of anti-PD-L1 antibody for treating sepsis (Hotchkiss et al., 2019). The patients received low-dose (10–100 mg) or high-dose (300–900 mg) treatment. The high-dose treatment significantly upregulated monocytic HLA-DR, and maintained this increase for over 28 days. There were no increased levels of cytokines (such as IL-6, IL-8, or IL-10). The study preliminarily verified the safety and potential effectiveness of anti-PD-L1 antibody/anti-PD-1 antibody treatment in sepsis patients with immunosuppression. However, the conclusions should be validated in phase II and III RCTs.

## Conclusion and Perspectives

**FIGURE 3:**
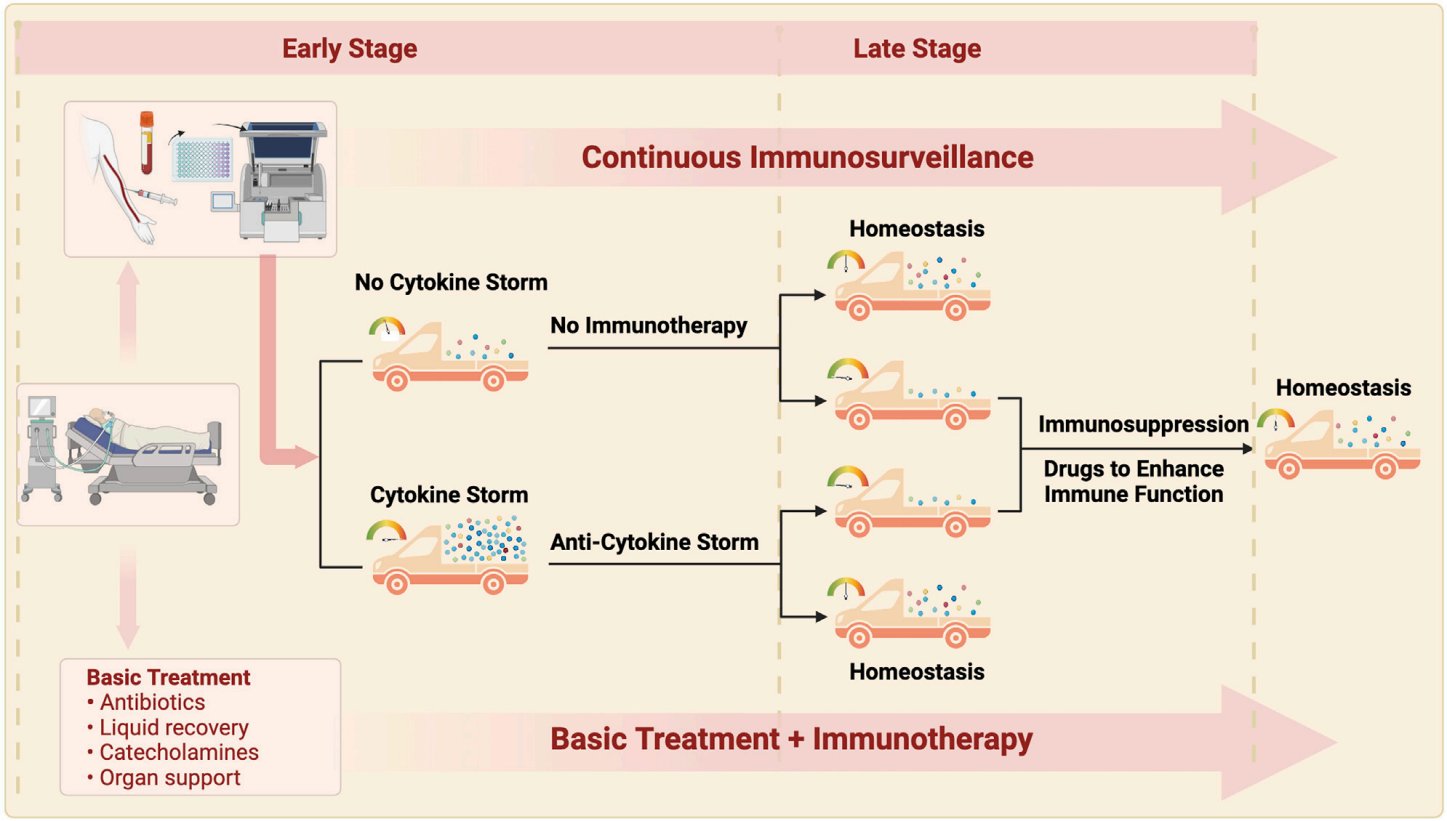
The ideal treatment for sepsis is routine treatment throughout the course of disease, continuous immunity monitoring, and moderate immunnity intervention. Patients with early sepsis are classified into two types: non-inflammatory storm and inflammatory storm according to the monitoring of immune indexes on admission: patients without inflammatory storm are given routine treatment, and appropriate anti-inflammatory storm therapy is adopted for patients with inflammatory storm. Some patients may achieve immune homeostasis after treatment improvement, and continuous immune monitoring found that the other patients may develope immunosuppression with the prolonged course of disease, so immunostimulation therapy is needed to finally restore the immune homeostasis. The goal of immunotherapy in sepsis is to maintain immune homeostasis by continuously monitoring the time of intervention, guiding the dose and course of intervention.

With the deepened understanding of sepsis, it has gradually been realized that the initially useful anti-infection treatment may not resolve all the problems. Due to the inherent complexity of the inflammatory response, in addition to ensuring the correct anti-infection treatment, therapeutic strategies for sepsis should also consider the patient’s basic immune status, pathogen-induced immunological changes, cytokine levels, and the endothelial protection and nutrition and metabolic support required. Recent studies have indicated that the immune changes caused by sepsis can seriously affect prognosis. Further in-depth research on the immune mechanisms underlying sepsis is crucial to therapeutic breakthroughs. It is reasonable to develop immune monitoring and evaluation techniques along with individualized treatment regimens for sepsis patients according to their individual immunological characteristics.The use of correct anti-infection treatment, fluid replacement, organ function support, timely use of immunomodulatory interventions, and development of accurate biomarkers to guide the clinical management (rather than relying only on clinical manifestations) might improve the treatment of patients with sepsis (Figure 3).

## References

[B1] Abraham E. Glauser M. P. Butler T. Garbino J. Gelmont D. Laterre P. F. (1997). p55 Tumor Necrosis Factor Receptor Fusion Protein in the Treatment of Patients with Severe Sepsis and Septic Shock. JAMA 277, 1531–1538. 10.1001/jama.277.19.153110.1001/jama.1997.03540430043031 9153367

[B2] Adhikari N. K. Fowler R. A. Bhagwanjee S. Rubenfeld G. D. (2010). Critical Care and the Global burden of Critical Illness in Adults. The Lancet 376, 1339–1346. 10.1016/S0140-6736(10)60446-1 PMC7136988 20934212

[B3] Aird W. C. (2003). The Role of the Endothelium in Severe Sepsis and Multiple Organ Dysfunction Syndrome. Blood 101, 3765–3777. 10.1182/blood-2002-06-1887 12543869

[B4] Alobaidi R. Basu R. K. Goldstein S. L. Bagshaw S. M. (2015). Sepsis-associated Acute Kidney Injury. Semin. Nephrol. 35, 2–11. 10.1016/j.semnephrol.2015.01.002 25795495 PMC4507081

[B5] Annane D. Renault A. Brun-Buisson C. Megarbane B. Quenot J.-P. Siami S. (2018). Hydrocortisone Plus Fludrocortisone for Adults with Septic Shock. N. Engl. J. Med. 378, 809–818. 10.1056/NEJMoa1705716 29490185

[B6] Ardura J. A. Rackov G. Izquierdo E. Alonso V. Gortazar A. R. Escribese M. M. (2019). Targeting Macrophages: Friends or Foes in Disease?. Front. Pharmacol. 10, 1255. 10.3389/fphar.2019.01255 31708781 PMC6819424

[B7] Bagshaw S. M. George C. Bellomo R. (2008). ANZICS Database Management Committee.Early Acute Kidney Injury and Sepsis: a Multicentre Evaluation. Crit. Care 12, R47. 10.1186/cc6863 18402655 PMC2447598

[B8] Bain J. M. Alonso M. F. Childers D. S. Walls C. A. Mackenzie K. Pradhan A. (2021). Immune Cells Fold and Damage Fungal Hyphae. Proc. Natl. Acad. Sci. USA 118, e2020484118. 10.1073/pnas.2020484118 33876755 PMC8053999

[B9] Behnes M. Bertsch T. Hoffmann U. (2013). TIMP-1 Gene Polymorphism: Are Genetics Able to Predict Outcome of Septic Patients?. Crit. Care 17, 170. 10.1186/cc12799 23890414 PMC4056612

[B10] Biswas S. K. Lopez-Collazo E. (2009). Endotoxin Tolerance: New Mechanisms, Molecules and Clinical Significance. Trends Immunol. 30, 475–487. 10.1016/j.it.2009.07.009 19781994

[B11] Bociąga-Jasik M. Cieśla A. Kalinowska-Nowak A. Skwara P. Garlicki A. Mach T. (2011). Role of IL-6 and Neopterin in the Pathogenesis of Herpetic Encephalitis. Pharmacol. Rep. 63, 1203–1209. 10.1016/s1734-1140(11)70640-5 22180363

[B12] Bone R. C. Balk R. A. Cerra F. B. Dellinger R. P. Fein A. M. Knaus W. A. (1992). Definitions for Sepsis and Organ Failure and Guidelines for the Use of Innovative Therapies in sepsisThe ACCP/SCCM Consensus Conference Committee. American College of Chest Physicians/Society of Critical Care Medicine. Chest 101, 1644–1655. 10.1378/chest.101.6.1644 1303622

[B13] Bone R. C. (1996). Toward a Theory Regarding the Pathogenesis of the Systemic Inflammatory Response Syndrome. Crit. Care Med. 24, 163–172. 10.1097/00003246-199601000-00026 8565523

[B14] Boomer J. S. To K. Chang K. C. Takasu O. Osborne D. F. Walton A. H. (2011). Immunosuppression in patients who die of sepsis and multiple organ failure. JAMA 306, 2594–2605. 10.1001/jama.2011.1829 22187279 PMC3361243

[B15] Boomer J. S. Shuherk-Shaffer J. Hotchkiss R. S. Green J. M. (2012). A Prospective Analysis of Lymphocyte Phenotype and Function over the Course of Acute Sepsis. Crit. Care 16, R112. 10.1186/cc11404 22742734 PMC3580670

[B16] Borriello F. Galdiero M. R. Varricchi G. Loffredo S. Spadaro G. Marone G. (2019). Innate Immune Modulation by GM-CSF and IL-3 in Health and Disease. Int. J. Mol. Sci. 20, 834. 10.3390/ijms20040834 PMC6412223 30769926

[B17] Bouras M. Asehnoune K. Roquilly A. (2018). Contribution of Dendritic Cell Responses to Sepsis-Induced Immunosuppression and to Susceptibility to Secondary Pneumonia. Front. Immunol. 9, 2590. 10.3389/fimmu.2018.02590 30483258 PMC6243084

[B18] Brahmamdam P. Inoue S. Unsinger J. Chang K. C. McDunn J. E. Hotchkiss R. S. (2010). Delayed Administration of Anti-PD-1 Antibody Reverses Immune Dysfunction and Improves Survival during Sepsis. J. Leukoc. Biol. 88, 233–240. 10.1189/jlb.0110037 20483923 PMC6607999

[B19] Brinkmann V. Reichard U. Goosmann C. Fauler B. Uhlemann Y. Weiss D. S. (2004). Neutrophil Extracellular Traps Kill Bacteria. Science 303, 1532–1535. 10.1126/science.1092385 15001782

[B20] Burke J. D. Young H. A. (2019). IFN-γ: A Cytokine at the Right Time, Is in the Right Place. Semin. Immunol. 43, 101280. 10.1016/j.smim.2019.05.002 31221552 PMC7367502

[B21] Camerini R. Garaci E. (2015). Historical Review of Thymosin α 1 in Infectious Diseases. Expert Opin. Biol. Ther. Null, S117–S127. 10.1517/14712598.2015.1033393 26098768

[B22] Campbell K. S. Hasegawa J. (2013). Natural Killer Cell Biology: an Update and Future Directions. J. Allergy Clin. Immunol. 132, 536–544. 10.1016/j.jaci.2013.07.006 23906377 PMC3775709

[B23] Cao Y. Chai Y. F. Deng Y. Fang B. J. Liu M. H. Lu Z. Q. (2018). Guidelines for Emergency Treatment of Sepsis/septic Shock in China. J. Clin. Emerg. 19, 567–588. 10.13201/j.issn.1009-5918.2018.09.001

[B24] Cavaillon J. Adib-Conquy M. (2006). Bench-to-bedside Review: Endotoxin Tolerance as a Model of Leukocyte Reprogramming in Sepsis. Crit. Care 10, 233. 10.1186/cc5055 17044947 PMC1751079

[B25] Cazalis M. Friggeri A. Cavé L. Demaret J. Barbalat V. Cerrato E. (2013). Decreased HLA-DR Antigen-Associated Invariant Chain (CD74) mRNA Expression Predicts Mortality after Septic Shock. Crit. Care 17, R287. 10.1186/cc13150 24321376 PMC4056003

[B26] Chan J. K. Roth J. Oppenheim J. J. Tracey K. J. Vogl T. Feldmann M. (2012). Alarmins: Awaiting a Clinical Response. J. Clin. Invest. 122, 2711–2719. 10.1172/JCI62423 22850880 PMC3408740

[B27] Chen T. Cao Q. Wang Y. Harris D. C. H. (2019). M2 Macrophages in Kidney Disease: Biology, Therapies, and Perspectives. Kidney Int. 95, 760–773. 10.1016/j.kint.2018.10.041 30827512

[B28] Christaki E. Anyfanti P. Opal S. M. (2011). Immunomodulatory Therapy for Sepsis: an Update. Expert Rev. Anti Infect. Ther. 9, 1013–1033. 10.1586/eri.11.122 22029521

[B29] Chu H. Zhou J. Wong B. H. Y. Li C. Cheng Z. S. Lin X. (2014). Productive Replication of Middle East Respiratory Syndrome Coronavirus in Monocyte-Derived Dendritic Cells Modulates Innate Immune Response. Virology 2014, 197–205. 10.1016/j.virol.2014.02.018 PMC7111975 24725946

[B30] Conti H. R. Gaffen S. L. (2015). IL-17-Mediated Immunity to the Opportunistic Fungal Pathogen Candida Albicans. J. Immunol. 195, 780–788. 10.4049/jimmunol.1500909 26188072 PMC4507294

[B31] Cossarizza A. Chang H-D. Radbruch A. Akdis M. Andrä I. Annunziato F. (2017). Guidelines for the Use of Flow Cytometry and Cell Sorting in Immunological Studies. Eur. J. Immunol. 47, 1584–1797. 10.1002/eji.201646632 29023707 PMC9165548

[B32] Coyne C. B. Bozym R. Morosky S. A. Hanna S. L. Mukherjee A. Tudor M. (2011). Comparative RNAi Screening Reveals Host Factors Involved in Enterovirus Infection of Polarized Endothelial Monolayers. Cell Host Microbe 9, 70–82. 10.1016/j.chom.2011.01.001 21238948 PMC3048761

[B33] Czaikoski P. G. Mota J. M. S. C. Nascimento D. C. Sônego F. Castanheira F. V. S. Melo P. H. (2016). Neutrophil Extracellular Traps Induce Organ Damage during Experimental and Clinical Sepsis. PLoS One 11, e0148142. 10.1371/journal.pone.0148142 26849138 PMC4743982

[B34] Davey M. S. Morgan M. P. Liuzzi A. R. Tyler C. J. Khan M. W. A. Szakmany T. (2014). Microbe-specific Unconventional T Cells Induce Human Neutrophil Differentiation into Antigen Cross-Presenting Cells. J. Immunol. 193, 3704–3716. 10.4049/jimmunol.1401018 25165152 PMC4169984

[B35] de Pablo R. Monserrat J. Torrijos C. Martín M. Prieto A. Alvarez-Mon M. (2012). The Predictive Role of Early Activation of Natural Killer Cells in Septic Shock. Crit. Care 16, 413. 10.1186/cc11204 22405329 PMC3681341

[B36] Delano M. J. Ward P. A. (2016). Sepsis-induced Immune Dysfunction: Can Immune Therapies Reduce Mortality?. J. Clin. Invest. 126, 23–31. 10.1172/JCI82224 26727230 PMC4701539

[B37] Demaret J. Venet F. Friggeri A. Cazalis M. Plassais J. Jallades L. (2015). Marked Alterations of Neutrophil Functions during Sepsis-Induced Immunosuppression. J. Leukoc. Biol. 98, 1081–1090. 10.1189/jlb.4A0415-168RR 26224052

[B38] Deutschman C. S. Tracey K. J. (2014). Sepsis: Current Dogma and New Perspectives. Immunity 40, 463–475. 10.1016/j.immuni.2014.04.001 24745331

[B39] Döcke W. D. Randow F. Syrbe U. Krausch D. Asadullah K. Reinke P. (1997). Monocyte Deactivation in Septic Patients: Restoration by IFN-Gamma Treatment. Nat. Med. 3, 678–681. 10.1038/nm0697-678 9176497

[B40] Dreschler K. Bratke K. Petermann S. Thamm P. Kuepper M. Virchow J. C. (2012). Altered Phenotype of Blood Dendritic Cells in Patients with Acute Pneumonia. Respiration 83, 209–217. 10.1159/000328406 21677425

[B41] Drifte G. Dunn-Siegrist I. Tissières P. Pugin J. (2013). Innate Immune Functions of Immature Neutrophils in Patients with Sepsis and Severe Systemic Inflammatory Response Syndrome. Crit. Care Med. 41, 820–832. 10.1097/CCM.0b013e318274647d 23348516

[B42] Drummond R. A. Lionakis M. S. (2016). Mechanistic Insights into the Role of C-type Lectin Receptor/CARD9 Signaling in Human Antifungal Immunity. Front Cel Infect Microbiol 6, 39. 10.3389/fcimb.2016.00039 PMC4820464 27092298

[B43] Faivre V. Lukaszewicz A. C. Alves A. Charron D. Payen D. Haziot A. (2012). Human Monocytes Differentiate into Dendritic Cells Subsets that Induce Anergic and Regulatory T Cells in Sepsis. PLoS One 7, e47209. 10.1371/journal.pone.0047209 23071758 PMC3468528

[B44] Faivre V. Lukaszewicz A-C. Alves A. Charron D. Payen D. Haziot A. (2007). Accelerated *In Vitro* Differentiation of Blood Monocytes into Dendritic Cells in Human Sepsis. Clin. Exp. Immunol. 147, 426–439. 10.1111/j.1365-2249.2006.03287.x 17302891 PMC1810505

[B45] Fei C. Xiaoyong X. He S. Yi S. (2015). Mechanism of Th17 in Immuneresponse to Aspergillus fumigatus Infection. Int. J. Respir. 35, 136–138. 10.3760/cma.j.issn.1673-436X.2015.02.013

[B46] Fisher C. J. Agosti J. M. Opal S. M. Lowry S. F. Balk R. A. Sadoff J. C. (1996). Treatment of Septic Shock with the Tumor Necrosis Factor receptor:Fc Fusion Protein. The Soluble TNF Receptor Sepsis Study Group. N. Engl. J. Med. 334, 1697–1702. 10.1056/NEJM199606273342603 8637514

[B47] Fitzgerald K. A. Rowe D. C. Barnes B. J. Caffrey D. R. Visintin A. Latz E. (2003). LPS-TLR4 Signaling to IRF-3/7 and NF-kappaB Involves the Toll Adapters TRAM and TRIF. J. Exp. Med. 198, 1043–1055. 10.1084/jem.20031023 14517278 PMC2194210

[B48] Fleischmann C. Scherag A. Adhikari N. K. J. Hartog C. S. Tsaganos T. Schlattmann P. (2016). Assessment of Global Incidence and Mortality of Hospital-Treated Sepsis. Current Estimates and Limitations. Am. J. Respir. Crit. Care Med. 193, 259–272. 10.1164/rccm.201504-0781OC 26414292

[B49] Forel J-M. Chiche L. Thomas G. Mancini J. Farnarier C. Cognet C. (2012). Phenotype and Functions of Natural Killer Cells in Critically-Ill Septic Patients. PLoS One 7, e50446. 10.1371/journal.pone.0050446 23236375 PMC3516510

[B50] Francois B. Jeannet R. Daix T. Walton A. H. Shotwell M. S. Unsinger J. (2018). Interleukin-7 Restores Lymphocytes in Septic Shock: the IRIS-7 Randomized Clinical Trial. JCI Insight 3, e98960. 10.1172/jci.insight.98960 PMC5922293 29515037

[B51] Geginat J. Paroni M. Maglie S. Alfen J. S. Kastirr I. Gruarin P. (2014). Plasticity of Human CD4 T Cell Subsets. Front. Immunol. 5, 630. 10.3389/fimmu.2014.00630 25566245 PMC4267263

[B52] Geissmann F. Manz M. G. Jung S. Sieweke M. H. Merad M. Ley K. (2010). Development of Monocytes, Macrophages, and Dendritic Cells. Science 327, 656–661. 10.1126/science.1178331 20133564 PMC2887389

[B53] Gentile L. F. Cuenca A. G. Efron P. A. Ang D. Bihorac A. McKinley B. A. (2012). Persistent Inflammation and Immunosuppression: a Common Syndrome and New Horizon for Surgical Intensive Care. J. Trauma Acute Care Surg. 72, 1491–1501. 10.1097/TA.0b013e318256e000 22695412 PMC3705923

[B54] Ghosh S. Karin M. (2002). Missing Pieces in the NF-kappaB Puzzle. Cell 109, S81–S96. 10.1016/s0092-8674(02)00703-1 11983155

[B55] Giacconi R. Costarelli L. Malavolta M. Piacenza F. Galeazzi R. Gasparini N. (2014). Association Among 1267 A/G HSP70-2, -308 G/A TNF-α Polymorphisms and Pro-inflammatory Plasma Mediators in Old ZincAge Population. Biogerontology 15, 65–79. 10.1007/s10522-013-9480-1 24243066

[B56] Giamarellos-Bourboulis E. J. Opal S. M. (2016). The Role of Genetics and Antibodies in Sepsis. Ann. Transl Med. 4, 328. 10.21037/atm.2016.08.63 27713886 PMC5050196

[B57] Gibson J. F. Johnston S. A. (2015). Immunity to Cryptococcus Neoformans and C. Gattii during Cryptococcosis. Fungal Genet. Biol. 78, 76–86. 10.1016/j.fgb.2014.11.006 25498576 PMC4503824

[B58] Gong T. Liu L. Jiang W. Zhou R. (2020). DAMP-sensing Receptors in Sterile Inflammation and Inflammatory Diseases. Nat. Rev. Immunol. 20, 95–112. 10.1038/s41577-019-0215-7 31558839

[B59] Grimaldi D. Louis S. Pène F. Sirgo G. Rousseau C. Claessens Y. E. (2011). Profound and Persistent Decrease of Circulating Dendritic Cells Is Associated with ICU-Acquired Infection in Patients with Septic Shock. Intensive Care Med. 37, 1438–1446. 10.1007/s00134-011-2306-1 21805160

[B60] Gringhuis S. I. Wevers B. A. Kaptein T. M. van Capel T. M. M. Theelen B. Boekhout T. (2011). Selective C-Rel Activation *via* Malt1 Controls Anti-fungal TH-17 Immunity by Dectin-1 and Dectin-2. PLOS Pathog. 7, e1001259. 10.1371/journal.ppat.1001259 21283787 PMC3024268

[B61] Guignant C. Lepape A. Huang X. Kherouf H. Denis L. Poitevin F. (2011). Programmed Death-1 Levels Correlate with Increased Mortality, Nosocomial Infection and Immune Dysfunctions in Septic Shock Patients. Crit. Care 15, R99. 10.1186/cc10112 21418617 PMC3219369

[B62] Guisset O. Dilhuydy M. Thiébaut R. Lefèvre J. Camou F. Sarrat A. (2007). Decrease in Circulating Dendritic Cells Predicts Fatal Outcome in Septic Shock. Intensive Care Med. 33, 148–152. 10.1007/s00134-006-0436-7 17091240

[B63] Guo Y. Luan L. Patil N. K. Wang J. Bohannon J. K. Rabacal W. (2017). IL-15 Enables Septic Shock by Maintaining NK Cell Integrity and Function. J. Immunol. 198, 1320–1333. 10.4049/jimmunol.1601486 28031340 PMC5263185

[B64] Guo Y. Patil N. K. Luan L. Bohannon J. K. Sherwood E. R. (2018). The Biology of Natural Killer Cells during Sepsis. Immunology 153, 190–202. 10.1111/imm.12854 29064085 PMC5765373

[B65] Heath W. R. Carbone F. R. (2001). Cross-presentation in Viral Immunity and Self-Tolerance. Nat. Rev. Immunol. 1, 126–134. 10.1038/35100512 11905820

[B66] Hein F. Massin F. Cravoisy-Popovic A. Barraud D. Levy B. Bollaert P-E. (2010). The Relationship between CD4+CD25+CD127- Regulatory T Cells and Inflammatory Response and Outcome during Shock States. Crit. Care 14, R19. 10.1186/cc8876 20156359 PMC2875534

[B67] Heming N. Sivanandamoorthy S. Meng P. Bounab R. Annane D. (2018). Immune Effects of Corticosteroids in Sepsis. Front. Immunol. 9, 1736. 10.3389/fimmu.2018.01736 30105022 PMC6077259

[B68] Ho J. Yu J. Wong S. H. Zhang L. Liu X. Wong W. T. (2016). Autophagy in Sepsis: Degradation into Exhaustion?. Autophagy 12, 1073–1082. 10.1080/15548627.2016.1179410 27172163 PMC4990998

[B69] Holub M. Klucková Z. Beneda B. Hobstová J. Huzicka I. Prazák J. (2000). Changes in Lymphocyte Subpopulations and CD3+/DR+ Expression in Sepsis. Clin. Microbiol. Infect. 6, 657–660. 10.1046/j.1469-0691.2000.00175.x 11284925

[B70] Hotchkiss R. S. Colston E. Yende S. Angus D. C. Moldawer L. L. Crouser E. D. (2019). Immune Checkpoint Inhibition in Sepsis: A Phase 1b Randomized, Placebo-Controlled, Single Ascending Dose Study of Antiprogrammed Cell Death-Ligand 1 Antibody (BMS-936559). Crit. Care Med. 47, 632–642. 10.1097/CCM.0000000000003685 30747773 PMC7254685

[B71] Hotchkiss R. S. Monneret G. Payen D. (2013). Sepsis-induced Immunosuppression: from Cellular Dysfunctions to Immunotherapy. Nat. Rev. Immunol. 13, 862–874. 10.1038/nri3552 24232462 PMC4077177

[B72] Hotchkiss R. S. Nicholson D. W. (2006). Apoptosis and Caspases Regulate Death and Inflammation in Sepsis. Nat. Rev. Immunol. 6, 813–822. 10.1038/nri1943 17039247

[B73] Hsiao H. B. Chou A. H. Lin S. I. Chen I. H. Lien S. P. Liu C. C. (2014). Toll-like Receptor 9-mediated protection of Enterovirus 71 Infection in Mice Is Due to the Release of Danger-Associated Molecular Patterns. J. Virol. 88, 11658–11670. 10.1128/JVI.00867-14 25078697 PMC4178751

[B74] Huang C. Wang Y. Li X. Ren L. Zhao J. Hu Y. (2020). Clinical Features of Patients Infected with 2019 Novel Coronavirus in Wuhan, China. Lancet 395, 497–506. 10.1016/S0140-6736(20)30183-5 31986264 PMC7159299

[B75] Huang P. Zhou Y. Liu Z. Zhang P. (2016). Interaction between ANXA1 and GATA-3 in Immunosuppression of CD4 T Cells. Mediators Inflamm. 2016, 1701059. 10.1155/2016/1701059 27833268 PMC5090097

[B76] Investigators. R. E. M. A. P-C. A. P. Gordon A. C. Mouncey P. R. Al-Beidh F. Rowan K. M. Nichol A. D. (2021). Interleukin-6 Receptor Antagonists in Critically Ill Patients with Covid-19. N. Engl. J. Med. 384, 1491–1502. 10.1056/NEJMoa2100433 33631065 PMC7953461

[B77] Iwasaki A. Medzhitov R. (2004). Toll-like Receptor Control of the Adaptive Immune Responses. Nat. Immunol. 5, 987–995. 10.1038/ni1112 15454922

[B78] Jacquin E. Apetoh L. (2018). Cell-Intrinsic Roles for Autophagy in Modulating CD4 T Cell Functions. Front. Immunol. 9, 1023. 10.3389/fimmu.2018.01023 29867990 PMC5954027

[B79] Kalvelage C. Zacharowski K. Bauhofer A. Gockel U. Adamzik M. Nierhaus A. (2019). Personalized Medicine with IgGAM Compared with Standard of Care for Treatment of Peritonitis after Infectious Source Control (The PEPPER Trial): Study Protocol for a Randomized Controlled Trial. Trials 20, 156. 10.1186/s13063-019-3244-4 30832742 PMC6399861

[B80] Kasten K. R. Muenzer J. T. Caldwell C. C. (2010). Neutrophils Are Significant Producers of IL-10 during Sepsis. Biochem. Biophys. Res. Commun. 393, 28–31. 10.1016/j.bbrc.2010.01.066 20097159 PMC2830356

[B81] Kawai T. Akira S. (2010). The Role of Pattern-Recognition Receptors in Innate Immunity: Update on Toll-like Receptors. Nat. Immunol. 11, 373–384. 10.1038/ni.1863 20404851

[B82] Keh D. Boehnke T. Weber-Cartens S. Schulz C. Ahlers O. Bercker S. (2003). Immunologic and Hemodynamic Effects of “Low-dose” Hydrocortisone in Septic Shock: a Double-Blind, Randomized, Placebocontrolled, Crossover Study. Am. J. Respir. Crit. Care Med. 167, 512–520. 10.1164/rccm.200205-446OC 12426230

[B83] Kjaergaard A. G. Nielsen J. S. Tønnesen E. Krog J. (2015). Expression of NK Cell and Monocyte Receptors in Critically Ill Patients-Ppotential Biomarkers of Sepsis. Scand. J. Immunol. 81, 249–258. 10.1111/sji.12272 25619264

[B84] Knaup H. Stahl K. Schmidt B. M. W. Idowu T. O. Busch M. Wiesner O. (2018). Early Therapeutic Plasma Exchange in Septic Shock: a Prospective Open-Label Nonrandomized Pilot Study Focusing on Safety, Hemodynamics, Vascular Barrier Function, and Biologic Markers. Crit. Care 22, 285. 10.1186/s13054-018-2220-9 30373638 PMC6206942

[B85] Kolaczkowska E. Kubes P. (2013). Neutrophil Recruitment and Function in Health and Inflammation. Nat. Rev. Immunol. 13, 159–175. 10.1038/nri3399 23435331

[B86] Kong S. L. Chui P. Lim B. Salto-Tellez M. (2009). Elucidating the Molecular Physiopathology of Acute Respiratory Distress Syndrome in Severe Acute Respiratory Syndrome Patients. Virus. Res. 145, 260–269. 10.1016/j.virusres.2009.07.014 19635508 PMC7114434

[B87] Kudo D. Kushimoto S. Miyagawa N. Sato T. Hasegawa M. Ito F. (2018). The Impact of Organ Dysfunctions on Mortality in Patients with Severe Sepsis: A Multicenter Prospective Observational Study. J. Crit. Care 45, 178–183. 10.1016/j.jcrc.2018.03.011 29549747

[B88] Kühlhorn F. Rath M. Schmoeckel K. Cziupka K. Nguyen H. H. Hildebrandt P. (2013). Foxp3+ Regulatory T Cells Are Required for Recovery from Severe Sepsis. PLoS One 8, e65109. 10.1371/journal.pone.0065109 23724126 PMC3665556

[B89] Kumar V. (2019). Natural Killer Cells in Sepsis: Underprivileged Innate Immune Cells. Eur. J. Cel Biol 98, 81–93. 10.1016/j.ejcb.2018.12.003 30583806

[B90] László I. Trásy D. Molnár Z. Fazakas J. (2015). Sepsis: From Pathophysiology to Individualized Patient Care. J. Immunol. Res. 2015, 510436. 10.1155/2015/510436 26258150 PMC4518174

[B91] Lawrence T. Natoli G. (2011). Transcriptional Regulation of Macrophage Polarization: Enabling Diversity with Identity. Nat. Rev. Immunol. 11, 750–761. 10.1038/nri3088 22025054

[B92] Leentjens J. Kox M. van der H. J. G. Netea M. G. Pickkers P. (2013). Immunotherapy for the Adjunctive Treatment of Sepsis: from Immunosuppression to Immunostimulation. Time for a Paradigm Change?. Am. J. Respir. Crit. Care Med. 187, 1287–1293. 10.1164/rccm.201301-0036CP 23590272

[B93] Lekkou A. Karakantza M. Mouzaki A. Kalfarentzos F. Gogos C. A. (2004). Cytokine Production and Monocyte HLA-DR Expression as Predictors of Outcome for Patients with Community-Acquired Severe Infections. Clin. Diagn. Lab. Immunol. 11, 161–167. 10.1128/cdli.11.1.161-167.2004 14715564 PMC321326

[B94] Leopold W. C. M. Hole C. R. Wozniak K. L. Wormley F. L. (2016). Cryptococcus and Phagocytes: Complex Interactions that Influence Disease Outcome. Front. Microbiol. 7, 105. 10.3389/fmicb.2016.00105 26903984 PMC4746234

[B95] Levy M. M. Fink M. P. Marshall J. C. Abraham E. Angus D. Cook D. (2003). 2001 SCCM/ESICM/ACCP/ATS/SIS International Sepsis Definitions Conference. Intensive Care Med. 29, 530–538. 10.1007/s00134-003-1662-x 12664219

[B96] Li Y. Ke J. Peng C. Wu F. Song Y. (2018). microRNA-300/NAMPT Regulates Inflammatory Responses through Activation of AMPK/mTOR Signaling Pathway in Neonatal Sepsis. Biomed. Pharmacother. 108, 271–279. 10.1016/j.biopha.2018.08.064 30223098

[B97] Lipinska-Gediga M. (2016). Coagulopathy in Sepsis - a New Look at an Old Problem. Anaesthesiol Intensive Ther. 48, 352–359. 10.5603/AIT.a2016.0051 27824218

[B98] Liu D. Yu Z. Yin J. Chen Y. Zhang H. Fan X. (2017). Effect of Ulinastatin Combined with Thymosin Alpha1 on Sepsis: A Systematic Review and Meta-Analysis of Chinese and Indian Patients. J. Crit. Care 39, 285–287. 10.1016/j.jcrc.2017.02.005 28283220

[B99] Liu J. Li S. Liu J. Liang B. Wang X. Wang H. (2020). Longitudinal Characteristics of Lymphocyte Responses and Cytokine Profiles in the Peripheral Blood of SARS-CoV-2 Infected Patients. EBioMedicine 55, 102763. 10.1016/j.ebiom.2020.102763 32361250 PMC7165294

[B100] Liu Y. C. Zou X. B. Chai Y. F. Yao Y. M. (2014). Macrophage Polarization in Inflammatory Diseases. Int. J. Biol. Sci. 10, 520–529. 10.7150/ijbs.8879 24910531 PMC4046879

[B101] Lobato-Pascual A. Saether P. C. Fossum S. Dissen E. Daws M. R. (2013). Mincle, the Receptor for Mycobacterial Cord Factor, Forms a Functional Receptor Complex with MCL and FcεRI-γ. Eur. J. Immunol. 43, 3167–3174. 10.1002/eji.201343752 23921530

[B102] Luan Y. Y. Yin C. F. Qin Q. H. Dong N. Zhu X. M. Sheng Z. Y. (2015). Effect of Regulatory T Cells on Promoting Apoptosis of T Lymphocyte and its Regulatory Mechanism in Sepsis. J. Interferon Cytokine Res. 35 (12), 969–980. 10.1089/jir.2014.0235 26309018 PMC4683547

[B103] Mansur A. Liese B. Steinau M. Ghadimi M. Bergmann I. Tzvetkov M. (2015). The CD14 Rs2569190 TT Genotype Is Associated with an Improved 30-Day Survival in Patients with Sepsis: A Prospective Observational Cohort Study. PLoS One 10, e0127761. 10.1371/journal.pone.0127761 26020644 PMC4447461

[B104] Manz M. G. Boettcher S. (2014). Emergency Granulopoiesis. Nat. Rev. Immunol. 14, 302. 10.1038/nri3660 24751955

[B105] Mao Z. R. Zhang S. L. Feng B. (2017). Association of IL-10 (-819T/C, -592A/C and -1082A/G) and IL-6 -174G/C Gene Polymorphism and the Risk of Pneumonia-Induced Sepsis. Biomarkers 22, 106–112. 10.1080/1354750X.2016.1210677 27388228

[B106] Marcos C. M. de Oliveira H. C. de Melo W. C. da Silva J. F. Assato P. A. Scorzoni L. (2016). Anti-immune Strategies of Pathogenic Fungi. Front Cel Infect Mi 6, 142. 10.3389/fcimb.2016.00142 PMC5108756 27896220

[B107] Matthay M. A. Ware L. B. Zimmerman G. A. (2012). The Acute Respiratory Distress Syndrome. J. Clin. Invest. 122, 2731–2740. 10.1172/JCI60331 22850883 PMC3408735

[B108] McDonald B. (2018). Neutrophils in Critical Illness. Cell Tissue Res 371, 607–615. 10.1007/s00441-017-2752-3 29247324

[B109] Meisel C. Schefold J. C. Pschowski R. Baumann T. Hetzger K. Gregor J. (2009). Granulocyte-macrophage colony-stimulating Factor to Reverse Sepsis-Associated Immunosuppression: a Double-Blind, Randomized, Placebo-Controlled Multicenter Trial. Am. J. Respir. Crit. Care Med. 180, 640–648. 10.1164/rccm.200903-0363OC 19590022

[B110] Mira J. C. Gentile L. F. Mathias B. J. Efron P. A. Brakenridge S. C. Mohr A. M. (2017). Sepsis Pathophysiology, Chronic Critical Illness, and Persistent Inflammation-Immunosuppression and Catabolism Syndrome. Crit. Care Med. 45, 253–262. 10.1097/CCM.0000000000002074 27632674 PMC5243156

[B111] Monneret G. Finck M. Venet F. Debard A. Bohé J. Bienvenu J. (2004). The Anti-inflammatory Response Dominates after Septic Shock: Association of Low Monocyte HLA-DR Expression and High Interleukin-10 Concentration. Immunol. Lett. 95, 193–198. 10.1016/j.imlet.2004.07.009 15388260

[B112] Mosser D. M. Zhang X. (2008). Interleukin-10: New Perspectives on an Old Cytokine. Immunol. Rev. 226, 205–218. 10.1111/j.1600-065X.2008.00706.x 19161426 PMC2724982

[B113] Murdock B. J. Teitz-Tennebaum S. Chen G. H. Dils A. J. Malachowski A. N. Curtis J. L. (2014). Early or Late IL-10 Blockade Enhances Th1 and Th17 Effector Responses and Promotes Fungal Clearance in Mice with Cryptococcal Lung Infection. JImmunol 193, 4107–4116. 10.4049/jimmunol.1400650 25225664 PMC4193595

[B114] Muszynski J. A. Nofziger R. Moore-Clingenpeel M. Greathouse K. Anglim L. Steele L. (2018). Early Immune Function and Duration of Organ Dysfunction in Critically III Children with Sepsis. Am. J. Respir. Crit. Care Med. 198, 361–369. 10.1164/rccm.201710-2006OC 29470918 PMC6835060

[B115] Nakahira K. Haspel J. A. Rathinam V. A. K. Lee S. J. Dolinay T. Lam H. C. (2011). Autophagy Proteins Regulate Innate Immune Responses by Inhibiting the Release of Mitochondrial DNA Mediated by the NALP3 Inflammasome. Nat. Immunol. 12, 222–230. 10.1038/ni.1980 21151103 PMC3079381

[B116] Netea M. G. Balkwill F. Chonchol M. Cominelli F. Donath M. Y. Giamarellos-Bourboulis E. J. (2017). A Guiding Map for Inflammation. Nat. Immunol. 18, 826–831. 10.1038/ni.3790 28722720 PMC5939996

[B117] Nierhaus A. Montag B. Timmler N. Frings D. P. Gutensohn K. Jung R. (2003). Reversal of Immunoparalysis by Recombinant Human Granulocyte-Macrophage colony-stimulating Factor in Patients with Severe Sepsis. Intensive Care Med. 29, 646–651. 10.1007/s00134-003-1666-6 12595977

[B118] Nomellini V. Kaplan L. J. Sims C. A. Caldwell C. C. (2018). Chronic Critical Illness and Persistent Inflammation: What Can We Learn from the Elderly, Injured, Septic, and Malnourished?. Shock 49, 4–14. 10.1097/SHK.0000000000000939 28682945

[B119] O'Neill L. A. Golenbock D. Bowie A. G. (2013). The History of Toll-like Receptors - Redefining Innate Immunity. Nat. Rev. Immunol. 13, 453–460. 10.1038/nri3446 23681101

[B120] Oami T. Watanabe E. Hatano M. Sunahara S. Fujimura L. Sakamoto A . (2017). Suppression of T Cell Autophagy Results in Decreased Viability and Function of T Cells through Accelerated Apoptosis in a Murine Sepsis Model Crit Care. Med 45, e77–e85. 10.1097/CCM.0000000000002016 PMC5364514 27618275

[B121] Oppert M. Schindler R. Husung C. Offermann K. Gräf K. J. Boenisch O. (2005). Low-dose Hydrocortisone Improves Shock Reversal and Reduces Cytokine Levels in Early Hyperdynamic Septic Shock. Crit. Care Med. 33, 2457–2464. 10.1097/01.ccm.0000186370.78639.23 16276166

[B122] Orozco H. Arch J. Medina-Franco H. Pantoja J. P. González Q. H. Vilatoba M. (2006). Molgramostim (GM-CSF) Associated with Antibiotic Treatment in Nontraumatic Abdominal Sepsis: a Randomized, Double-Blind, Placebo-Controlled Clinical Trial. Arch. Surg. 141, 150–153. 10.1001/archsurg.141.2.150 16490891

[B123] Ostrop J. Lang R. (2017). Contact, Collaboration, and Conflict: Signal Integration of Syk-Coupled C-type Lectin Receptors. J. Immunol. 198, 1403–1414. 10.4049/jimmunol.1601665 28167651

[B124] Osuchowski M. F. Craciun F. Weixelbaumer K. M. Duffy D. E. R. Remick D. G. (2012). Sepsis Chronically in MARS: Systemic Cytokine Responses Are Always Mixed Regardless of the Outcome, Magnitude, or Phase of Sepsis. J. Immunol. 189, 4648–4656. 10.4049/jimmunol.1201806 23008446 PMC3478412

[B125] Özkan H. Köksal N. Çetinkaya M. Kiliç Ş. Çelebi S. Oral B. (2012). Serum Mannose-Binding Lectin (MBL) Gene Polymorphism and Low MBL Levels Are Associated with Neonatal Sepsis and Pneumonia. J. Perinatol 32, 210–217. 10.1038/jp.2011.79 21681178

[B126] Padro D. C. Luong A. (2016). Innate Lymphoid Cells: The Innate Counterpart to T Helper Cells. Adv. Otorhinolaryngol. 79, 58–68. 10.1159/000445130 27466847

[B127] Panacek E. A. Marshall J. C. Albertson T. E. Johnson D. H. Johnson S. MacArthur R. D. (2004). Efficacy and Safety of the Monoclonal Anti-tumor Necrosis Factor Antibody F(ab')2 Fragment Afelimomab in Patients with Severe Sepsis and Elevated Interleukin-6 Levels. Crit. Care Med. 32, 2173–2182. 10.1097/01.ccm.0000145229.59014.6c 15640628

[B128] Park S. Y. Shrestha S. Youn Y. J. Kim J. K. Kim S. Y. Kim H. J. (2017). Autophagy Primes Neutrophils for Neutrophil Extracellular Trap Formation during Sepsis. Am. J. Respir. Crit. Care Med. 196, 577–589. 10.1164/rccm.201603-0596OC 28358992

[B129] Peiseler M. Kubes P. (2018). Macrophages Play an Essential Role in Trauma-Induced Sterile Inflammation and Tissue Repair. Eur. J. Trauma Emerg. Surg. 44, 335–349. 10.1007/s00068-018-0956-1 29666944

[B130] Pillay J. Kamp V. M. van H. E. Visser T. Tak T. Lammers J. W. (2012). A Subset of Neutrophils in Human Systemic Inflammation Inhibits T Cell Responses through Mac-1. J. Clin. Invest. 122, 327–336. 10.1172/JCI57990 22156198 PMC3248287

[B131] Pinder E. M. Rostron A. J. Hellyer T. P. Ruchaud-Sparagano M. H. Scott J. Macfarlane J. G. (2018). Randomised Controlled Trial of GM-CSF in Critically Ill Patients with Impaired Neutrophil Phagocytosis. Thorax 73, 918–925. 10.1136/thoraxjnl-2017-211323 30064991 PMC6166597

[B132] Pradeu T. Cooper E. L. (2012). The Danger Theory: 20 Years Later. Front. Immunol. 3, 287. 10.3389/fimmu.2012.00287 23060876 PMC3443751

[B133] Presneill J. J. Harris T. Stewart A. G. Cade J. F. Wilson J. W. (2002). A Randomized Phase II Trial of Granulocyte-Macrophage colony-stimulating Factor Therapy in Severe Sepsis with Respiratory Dysfunction. Am. J. Respir. Crit. Care Med. 166, 138–143. 10.1164/rccm.2009005 12119223

[B134] Qiu P. Liu Y. Zhang J. (2019). Review: the Role and Mechanisms of Macrophage Autophagy in Sepsis. Inflammation 42, 6–19. 10.1007/s10753-018-0890-8 30194660

[B135] Raetz C. R. H. Whitfield C. (2002). Lipopolysaccharide Endotoxins. Annu. Rev. Biochem. 71, 635–700. 10.1146/annurev.biochem.71.110601.135414 12045108 PMC2569852

[B136] RECOVERY Collaborative Group (2021). Tocilizumab in Patients Admitted to Hospital with COVID-19 (RECOVERY): a Randomised, Controlled, Open-Label, Platform Trial. Lancet 397 (10285), 1637–1645. 10.1016/S0140-6736(21)00676-0 33933206 PMC8084355

[B137] Retsas T. Huse K. Lazaridis L-D. Karampela N. Bauer M. Platzer M. (2018). Haplotypes Composed of Minor Frequency Single Nucleotide Polymorphisms of the TNF Gene Protect from Progression into Sepsis: A Study Using the New Sepsis Classification. Int. J. Infect. Dis. 67, 102–106. 10.1016/j.ijid.2017.12.008 29274398

[B138] Rhee C. Dantes R. Epstein L. Murphy D. J. Seymour C. W. Iwashyna T. J. (2017). Incidence and Trends of Sepsis in US Hospitals Using Clinical vs Claims Data, 2009-2014. JAMA 318, 1241–1249. 10.1001/jama.2017.13836 28903154 PMC5710396

[B139] Rhodes A. Evans L. E. Alhazzani W. Levy M. M. Antonelli M. Ferrer R. (2017). Surviving Sepsis Campaign: International Guidelines for Management of Sepsis and Septic Shock. Intensive Care Med. 43, 304–377. 10.1007/s00134-017-4683-6 28101605

[B140] Richer M. J. Lavallée D. J. Shanina I. Horwitz M. (2009). Toll-like Receptor 3 Signaling on Macrophages Is Required for Survival Following Coxsackievirus B4 Infection. PLoS One 4, e4127. 10.1371/journal.pone.0004127 19122812 PMC2606033

[B141] Ronco C. Tetta C. Mariano F. Wratten M. L. Bonello M. Bordoni V. (2003). Interpreting the Mechanisms of Continuous Renal Replacement Therapy in Sepsis: the Peak Concentration Hypothesis. Artif. Organs 27, 792–801. 10.1046/j.1525-1594.2003.07289.x 12940901

[B142] Roquilly A. McWilliam H. E. G. Jacqueline C. Tian Z. Cinotti R. Rimbert M. (2017). Local Modulation of Antigen-Presenting Cell Development after Resolution of Pneumonia Induces Long-Term Susceptibility to Secondary Infections. Immunity 47, 135–147. 10.1016/j.immuni.2017.06.021 28723546

[B143] Saitoh T. Fujita N. Jang M. H. Uematsu S. Yang B. G. Satoh T. (2008). Loss of the Autophagy Protein Atg16L1 Enhances Endotoxin-Induced IL-1beta Production. Nature 456, 264–268. 10.1038/nature07383 18849965

[B144] Sakr Y. Lobo S. M. Moreno R. P. Gerlach H. Ranieri V. M. Michalopoulos A. (2012). Patterns and Early Evolution of Organ Failure in the Intensive Care Unit and Their Relation to Outcome. Crit. Care 16, R222. 10.1186/cc11868 23158219 PMC3672601

[B145] Schwulst S. J. Muenzer J. T. Peck-Palmer O. M. Chang K. C. Davis C. G. McDonough J. S. (2008). Bim siRNA Decreases Lymphocyte Apoptosis and Improves Survival in Sepsis. Shock 30, 127–134. 10.1097/shk.0b013e318162cf17 18197142

[B146] Scumpia P. O. Delano M. J. Kelly K. M. O'Malley K. A. Efron P. A. McAuliffe P. F. (2006). Increased Natural CD4+CD25+ Regulatory T Cells and Their Suppressor Activity Do Not Contribute to Mortality in Murine Polymicrobial Sepsis. J. Immunol. 177, 7943–7949. 10.4049/jimmunol.177.11.7943 17114466

[B147] Shafiani S. Tucker-H. G. Kariyone A. Takatsu K. Urdahl K. B. (2010). Pathogen-specific Regulatory T Cells Delay the Arrival of Effector T Cells in the Lung during Early Tuberculosis. J. Exp. Med. 207, 1409–1420. 10.1084/jem.20091885 20547826 PMC2901066

[B148] Shao R. Fang Y. Yu H. Zhao L. Jiang Z. Li C. S. (2016). Monocyte Programmed Death Ligand-1 Expression after 3-4 Days of Sepsis Is Associated with Risk Stratification and Mortality in Septic Patients: a Prospective Cohort Study. Crit. Care 20, 124. 10.1186/s13054-016-1301-x 27156867 PMC4860759

[B149] Shen X. Cao K. Jiang J. Guan W. Du J. (2017). Neutrophil Dysregulation during Sepsis: an Overview and Update. J. Cel Mol Med 21, 1687–1697. 10.1111/jcmm.13112 PMC5571534 28244690

[B150] Sherwood E. R. Enoh V. T. Murphey E. D. Lin C. Y. (2004). Mice Depleted of CD8+ T and NK Cells Are Resistant to Injury Caused by Cecal Ligation and Puncture. Lab. Invest. 84, 1655–1665. 10.1038/labinvest.3700184 15448711

[B151] Sherwood E. R. Lin C. Y. Tao W. Hartmann C. A. Dujon J. E. French A. J. (2003). Beta 2 Microglobulin Knockout Mice Are Resistant to Lethal Intraabdominal Sepsis. Am. J. Respir. Crit. Care Med. 167, 1641–1649. 10.1164/rccm.200208-950OC 12626348

[B152] Shindo Y. Fuchs A. G. Davis C. G. Eitas T. Unsinger J. Burnham C-A. D. (2017). Interleukin 7 Immunotherapy Improves Host Immunity and Survival in a Two-Hit Model of *Pseudomonas aeruginosa* Pneumonia. J. Leukoc. Biol. 101, 543–554. 10.1189/jlb.4A1215-581R 27630218 PMC5235902

[B153] Short K. R. Kroeze E. J. B. V. Fouchier R. A. M. Kuiken T. (2014). Pathogenesis of Influenza-Induced Acute Respiratory Distress Syndrome. Lancet Infect. Dis. 14, 57–69. 10.1016/S1473-3099(13)70286-X 24239327

[B154] Singer M. Deutschman C. S. Seymour C. W. Shankar-Hari M. Annane D. Bauer Michael. (2016). The Third International Consensus Definitions for Sepsis and Septic Shock (Sepsis-3). JAMA 315, 801–810. 10.1001/jama.2016.0287 26903338 PMC4968574

[B155] Steinman R. M. Banchereau J. (2007). Taking Dendritic Cells into Medicine. Nature 449, 419–426. 10.1038/nature06175 17898760

[B156] Strother R. K. Danahy D. B. Kotov D. I. Kucaba T. A. Zacharias Z. R. Griffith T. S. (2016). Polymicrobial Sepsis Diminishes Dendritic Cell Numbers and Function Directly Contributing to Impaired Primary CD8 T Cell Responses *In Vivo* . J. Immunol. 197, 4301–4311. 10.4049/jimmunol.1601463 27798171 PMC5123856

[B157] Taeb A. M. Hooper M. H. Marik P. E. (2017). Sepsis: Current Definition, Pathophysiology, Diagnosis, and Management. Nutr. Clin. Pract. 32, 296–308. 10.1177/0884533617695243 28537517

[B158] Takatani Y. Ono K. Suzuki H. Inaba M. Sawada M. Matsuda N. (2018). Inducible Nitric Oxide Synthase during the Late Phase of Sepsis Is Associated with Hypothermia and Immune Cell Migration. Lab. Invest. 98, 629–639. 10.1038/s41374-018-0021-z 29449632

[B159] Takeuchi O. Akira S. (2010). Pattern Recognition Receptors and Inflammation. Cell 140, 805–820. 10.1016/j.cell.2010.01.022 20303872

[B160] Tao G. F. Zhang S. S. Zhu L. Y. Li W. Liu W. Z. (2017). Pharmacological Mech- Anism and Clinical Application of Ulinastatin. J. China Pharm. 28, 5020–5023. 10.6039/j.issn.1001-0408.2017.35.34

[B161] Tao W. Sherwood E. R. (2004). Beta2-microglobulin Knockout Mice Treated with Anti-asialoGM1 Exhibit Improved Hemodynamics and Cardiac Contractile Function during Acute Intra-abdominal Sepsis. Am. J. Physiol. Regul. Integr. Comp. Physiol. 286, R569–R575. 10.1152/ajpregu.00470.2003 14630624

[B162] Tatura R. Zeschnigk M. Hansen W. Steinmann J. Vidigal P. G. Hutzler M. (2015). Relevance of Foxp3⁺ Regulatory T Cells for Early and Late Phases of Murine Sepsis. Immunology 146, 144–156. 10.1111/imm.12490 26059660 PMC4552509

[B163] Taudien S. Lausser L. Giamarellos-Bourboulis E. J. Sponholz C. Schöneweck F. Felder M. (2016). Genetic Factors of the Disease Course after Sepsis: Rare Deleterious Variants Are Predictive. EBioMedicine 12, 227–238. 10.1016/j.ebiom.2016.08.037 27639823 PMC5078585

[B164] Tavares-Murta B. M. Zaparoli M. Ferreira R. B. Silva-Vergara M. L. Oliveira C. H. B. Murta E. F. C. (2002). Failure of Neutrophil Chemotactic Function in Septic Patients. Crit. Care Med. 30, 1056–1061. 10.1097/00003246-200205000-00017 12006803

[B165] Thompson C. M. Holden T. D. Rona G. Laxmanan B. Black R. A. OʼKeefe G. E. (2014). Toll-like Receptor 1 Polymorphisms and Associated Outcomes in Sepsis after Traumatic Injury: a Candidate Gene Association Study. Ann. Surg. 259, 179–185. 10.1097/SLA.0b013e31828538e8 23478521 PMC3686843

[B166] Tian F. Han Y. Song J. Lei J. Yan X. Xie N. (2016). Pulmonary Resident Neutrophils Regulate the Production of GM-CSF and Alveolar Macrophages. FEBS J. 283, 1465–1474. 10.1111/febs.13684 26881904

[B167] Triantafilou K. Orthopoulos G. Vakakis E. Ahmed Mohamed A. E. Golenbock D. T. Lepper P. M. (2005). Human Cardiac Inflammatory Responses Triggered by Coxsackie B Viruses Are Mainly Toll-like Receptor (TLR) 8-dependent. Cell Microbiol 7, 1117–1126. 10.1111/j.1462-5822.2005.00537.x 16008579

[B168] Tsao C. M. Ho S. T. Wu C. C. (2015). Coagulation Abnormalities in Sepsis. Acta Anaesthesiol Taiwan 53, 16–22. 10.1016/j.aat.2014.11.002 25544351

[B169] van der B. R. Nijhuis L. Pervolaraki K. Compeer E. B. Jongeneel L. H. van Gijn M. (2014). Defects in Mitochondrial Clearance Predispose Human Monocytes to Interleukin-1β Hypersecretion. J. Biol. Chem. 289, 5000–5012. 10.1074/jbc.M113.536920 24356959 PMC3931060

[B170] van der P. T. van de V. F. L. Scicluna B. P. Netea M. G. (2017). The Immunopathology of Sepsis and Potential Therapeutic Targets. Nat. Rev. Immunol. 17, 407–420. 10.1038/nri.2017.36 28436424

[B171] Vaure C. Liu Y. (2014). A Comparative Review of Toll-like Receptor 4 Expression and Functionality in Different Animal Species. Front. Immunol. 5, 316. 10.3389/fimmu.2014.00316 25071777 PMC4090903

[B172] Venet F. Monneret G. (2018). Advances in the Understanding and Treatment of Sepsis-Induced Immunosuppression. Nat. Rev. Nephrol. 14, 121–137. 10.1038/nrneph.2017.165 29225343

[B173] Vincent J. L. Moreno R. Takala J. Willatts S. De Mendonça A. Bruining H. (1996). The SOFA (Sepsis-Related Organ Failure Assessment) Score to Describe Organ Dysfunction/failure. On Behalf of the Working Group on Sepsis-Related Problems of the European Society of Intensive Care Medicine. Intensive Care Med. 22, 707–710. 10.1007/BF01709751 8844239

[B174] Vincent J. L. Sakr Y. Sprung C. L. Ranieri V. M. Reinhart K. Gerlach H. (2006). Sepsis in European Intensive Care Units: Results of the SOAP Study. Crit. Care Med. 34, 344–353. 10.1097/01.ccm.0000194725.48928.3a 16424713

[B175] Volk H. D. Reinke P. Krausch D. Zuckermann H. Asadullah K. Müller J. M. (1996). Monocyte Deactivation-Rrationale for a New Therapeutic Strategy in Sepsis. Intensive Care Med. 22, S474–S481. 10.1007/BF01743727 8923092

[B176] Weber S. U. Schewe J. C. Lehmann L. E. Müller S. Book M. Klaschik S. (2008). Induction of Bim and Bid Gene Expression during Accelerated Apoptosis in Severe Sepsis. Crit. Care 12, R128. 10.1186/cc7088 18925930 PMC2592767

[B177] Wen H. Dou Y. Hogaboam C. M. Kunkel S. L. (2008). Epigenetic Regulation of Dendritic Cell-Derived Interleukin-12 Facilitates Immunosuppression after a Severe Innate Immune Response. Blood 111, 1797–1804. 10.1182/blood-2007-08-106443 18055863 PMC2234040

[B178] Werdan K. Pilz G. Müller-Werdan U. Maas Enriquez M. Schmitt D. V. Mohr F. W. (2008). Immunoglobulin G Treatment of Postcardiac Surgery Patients with Score-Identified Severe Systemic Inflammatory Response Syndrome-Tthe ESSICS Study. Crit. Care Med. 36, 716–723. 10.1097/01.CCM.0B013E3181611F62F 18091548

[B179] Werdan l. Pilz G. Bujdoso O. Fraunberger P. Neeser G. Schmieder R. E. (2007). Score-based Immunoglobulin G Therapy of Patients with Sepsis: the SBITS Study. Crit. Care Med. 35, 2693–2701. 10.1097/01.ccm.0000295426.37471.79 18074471

[B180] Whitney P. G. Bär E. Osorio F. Rogers N. C. Schraml B. U. Deddouche S. (2014). Syk Signaling in Dendritic Cells Orchestrates Innate Resistance to Systemic Fungal Infection. Plos Pathog. 10, e1004276. 10.1371/journal.ppat.1004276 25033445 PMC4102599

[B181] Wolk K. Döcke W. D. von Baehr V. Volk H. D. Sabat R. (2000). Impaired Antigen Presentation by Human Monocytes during Endotoxin Tolerance. Blood 96, 218–223. 10891454

[B182] Wong C. K. Lam C. W. K. Wu A. K. L. Ip W. K. Lee N. L. S. Chan I. H. S. (2004). Plasma Inflammatory Cytokines and Chemokines in Severe Acute Respiratory Syndrome. Clin. Exp. Immunol. 136, 95–103. 10.1111/j.1365-2249.2004.02415.x 15030519 PMC1808997

[B183] Wu D. D. Li T. Ji X. Y. (2017). Dendritic Cells in Sepsis: Pathological Alterations and Therapeutic Implications. J. Immunol. Res. 2017, 3591248. 10.1155/2017/3591248 29075648 PMC5624156

[B184] Wu J. Zhou L. Liu J. Ma G. Kou Q. He Z. (2013). The Efficacy of Thymosin Alpha 1 for Severe Sepsis (ETASS): a Multicenter, Single-Blind, Randomized and Controlled Trial. Crit. Care 17, R8. 10.1186/cc11932 23327199 PMC4056079

[B185] Wu M. Ji J. J. Zhong L. Shao Z. Y. Xie Q. F. Liu Z. Y. (2020). Thymosin α1 Therapy in Critically Ill Patients with COVID-19: A Multicenter Retrospective Cohort Study. Int. Immunopharmacol 88, 106873. 10.1016/j.intimp.2020.106873 32795897 PMC7409727

[B186] Wu Z. Yao Y. Hong G. Xu X. Liu Y. Dong N. (2014). Role of Mitofusin-2 in High Mobility Group Box-1 Protein-Mediated Apoptosis of T Cells *In Vitro* . Cell Physiol Biochem 33, 769–783. 10.1159/000358651 24662494

[B187] Xie J. Chen C-W. Sun Y. Laurie S. J. Zhang W. Otani S. (2019). Increased Attrition of Memory T Cells during Sepsis Requires 2B4. JCI Insight 4, e126030. 10.1172/jci.insight.126030 PMC6538360 31045575

[B188] Xie J. Wang H. Kang Y. Zhou L. Liu Z. Qin B. (2020). The Epidemiology of Sepsis in Chinese ICUs: A National Cross-Sectional Survey. Crit. Care Med. 48, e209–e218. 10.1097/CCM.0000000000004155 31804299

[B189] Xu Q. Yan Q. Chen S. (2018). Ulinastatin Is Effective in Reducing Mortality for Critically Ill Patients with Sepsis: a Causal Mediation Analysis. Sci. Rep. 8, 14360. 10.1038/s41598-018-32533-9 30254204 PMC6156583

[B190] Xu X. Han M. Li T. Sun W. Wang D. Fu B. (2020). Effective Treatment of Severe COVID-19 Patients with Tocilizumab. Proc. Natl. Acad. Sci. U S A. 117, 10970–10975. 10.1073/pnas.2005615117 32350134 PMC7245089

[B191] Yamamoto M. Sato S. Mori K. Hoshino K. Takeuchi O. Takeda K. (2002). Cutting Edge: a Novel Toll/IL-1 Receptor Domain-Containing Adapter that Preferentially Activates the IFN-Beta Promoter in the Toll-like Receptor Signaling. J. Immunol. 169, 6668–6672. 10.4049/jimmunol.169.12.6668 12471095

[B192] Yang X. M. Han G. C. Ma Y. F. Li Y. (2013). Research Progress in Immunoregulatory Role of Macrophages in Sepsis. Mil. Med. Sci. 37, 469–471. 10.7644/j.issn.1674-9960.2013.06.019

[B193] Yoon S. J. Kim S. J. Lee S. M. (2017). Overexpression of HO-1 Contributes to Sepsis-Induced Immunosuppression by Modulating the Th1/Th2 Balance and Regulatory T-Cell Function. J. Infect. Dis. 215, 1608–1618. 10.1093/infdis/jix142 28368519

[B194] Zhang D. Zhou J. Ye L. C. Li J. Wu Z. Li Y. (2018). Autophagy Maintains the Integrity of Endothelial Barrier in LPS-Induced Lung Injury. J. Cel Physiol 233, 688–698. 10.1002/jcp.25928 28328069

[B195] Zhang S. Y. Herman M. Ciancanelli M. J. Pérez de Diego R. Sancho-Shimizu V. Abel L. (2013). TLR3 Immunity to Infection in Mice and Humans. Curr. Opin. Immunol. 25, 19–33. 10.1016/j.coi.2012.11.001 23290562 PMC3594520

[B196] Zhao G. Yao Y. Lu Z. Hong G. Zhu X. Wu Y. (2012). Up-regulation of Mitofusin-2 Protects CD4+ T Cells from HMGB1-Mediated Immune Dysfunction Partly through Ca(2+)-NFAT Signaling Pathway. Cytokine 59, 79–85. 10.1016/j.cyto.2012.03.026 22549180

[B197] Zhou J. Chu H. Li C. Wong B. H. Y. Cheng Z. S. Poon V. K. M. (2014). Active Replication of Middle East Respiratory Syndrome Coronavirus and Aberrant Induction of Inflammatory Cytokines and Chemokines in Human Macrophages: Implications for Pathogenesis. J. Infect. Dis. 209, 1331–1342. 10.1093/infdis/jit504 24065148 PMC7107356

[B198] Zhu L. L. Zhao X. Q. Jiang C. Y. You Y. Chen X. P. Jiang Y. Y. (2013). C-type Lectin Receptors Dectin-3 and Dectin-2 Form a Heterodimeric Pattern-Recognition Receptor for Host Defense against Fungal Infection. Immunity 39, 324–334. 10.1016/j.immuni.2013.05.017 23911656

